# Mating-Induced Increase in Germline Stem Cells via the Neuroendocrine System in Female *Drosophila*

**DOI:** 10.1371/journal.pgen.1006123

**Published:** 2016-06-16

**Authors:** Tomotsune Ameku, Ryusuke Niwa

**Affiliations:** 1 Graduate School of Life and Environmental Sciences, University of Tsukuba, Tsukuba, Ibaraki, Japan; 2 Faculty of Life and Environmental Sciences, University of Tsukuba, Tsukuba, Ibaraki, Japan; 3 PRESTO, Japan Science and Technology Agency, Kawaguchi, Saitama, Japan; Katholieke Universiteit Leuven, BELGIUM

## Abstract

Mating and gametogenesis are two essential components of animal reproduction. Gametogenesis must be modulated by the need for gametes, yet little is known of how mating, a process that utilizes gametes, may modulate the process of gametogenesis. Here, we report that mating stimulates female germline stem cell (GSC) proliferation in *Drosophila melanogaster*. Mating-induced increase in GSC number is not simply owing to the indirect effect of emission of stored eggs, but rather is stimulated by a male-derived Sex Peptide (SP) and its receptor SPR, the components of a canonical neuronal pathway that induces a post-mating behavioral switch in females. We show that ecdysteroid, the major insect steroid hormone, regulates mating-induced GSC proliferation independently of insulin signaling. Ovarian ecdysteroid level increases after mating and transmits its signal directly through the ecdysone receptor expressed in the ovarian niche to increase the number of GSCs. Impairment of ovarian ecdysteroid biosynthesis disrupts mating-induced increase in GSCs as well as egg production. Importantly, feeding of ecdysteroid rescues the decrease in GSC number caused by impairment of neuronal SP signaling. Our study illustrates how female GSC activity is coordinately regulated by the neuroendocrine system to sustain reproductive success in response to mating.

## Author Summary

In many animals, gametogenesis is supported by germline stem cells (GSCs). Since GSCs are the fundamental cell population for successful reproduction, how such special GSCs are precisely proliferated is a long-standing question in biology. In the fruit fly *Drosophila melanogaster*, it is known that gametogenesis is affected by environmental conditions and external stimuli. However, it remains unclear how GSC proliferation is regulated in response to such external signals at the molecular and cellular levels. Here, we report that mating stimulates stem cell activity in female ovary. Mating induces GSC proliferation via neuronal signaling stimulated by a male-derived Sex Peptide (SP). SP-dependent GSC proliferation is controlled by the ecdysteroid, the major insect steroid hormone, independently of insulin signaling. A significance of our study is to illustrate a role of the neuroendocrine regulatory mechanism in the GSC proliferation after mating, which might contribute to continuously and adaptively producing eggs. Furthermore, our findings will open the door to a new understanding of an indispensable mechanistic link between animal behavior (mating) and the regulation of stem cells.

## Introduction

Gametogenesis is modulated by stimuli from environments surrounding individual organisms. One such example is mating stimulus, which accelerates egg production [1]. Because mating is a process that utilizes many eggs, it is plausible that mating may modulate the process of gametogenesis. However, it remains largely unclear how mating affects gametogenesis at a cellular level.

**Fig 1:**
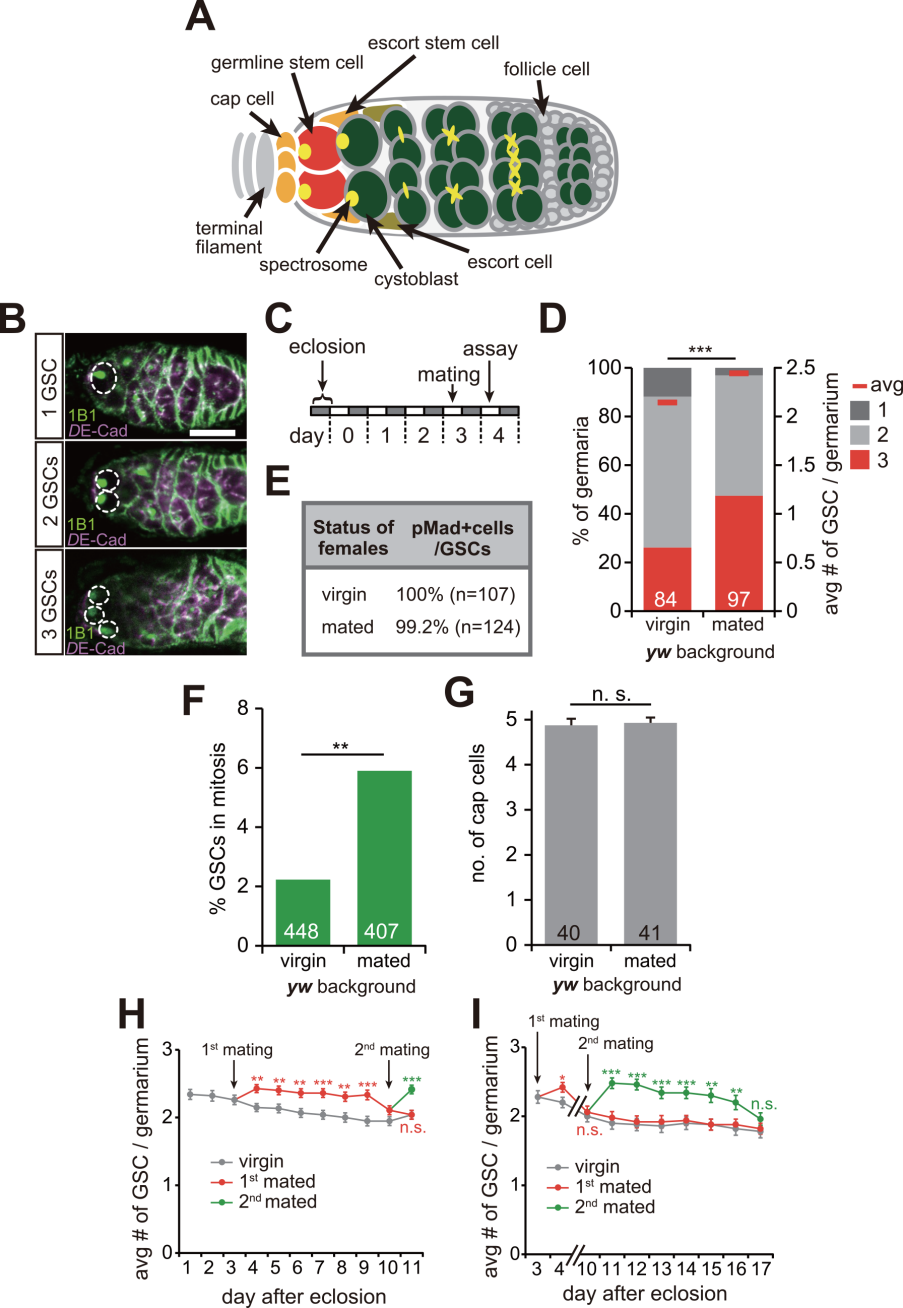
Mating stimulates GSC proliferation. (A) *Drosophila* germarium. GSCs (red) reside in a niche, comprising somatic cells such as cap cells (orange), terminal filament, and escort stem cells. GSCs are identifiable by their typical spectrosome morphology (yellow) and their location (adjacent to the cap cells). GSC division produces one self-renewing daughter and one cystoblast that differentiates into a germline cyst. (B) Representative examples of the germaria of wild-type flies containing one, two, or three GSCs in each germaria. Samples were stained with 1B1 (green) and anti-*D*E-cadherin (magenta) antibodies, which visualized GSCs (dotted circles) and overall cell membranes, respectively. (C) Protocol for GSC analysis for all experiments in this study. 3-day-old females were mated with males and used for assay 1 day after mating. (D) Frequencies of germaria containing one, two, and three GSCs (left y axis), and average number of GSCs per germarium (right y axis) in virgin and mated females in wild-type flies. Mated females showed increased GSC numbers as compared with virgin females. (E) Frequencies of GSC expressing phosphorylated Mad (pMad), which is a stem cell marker [4], were almost the same in mated female flies as compared with virgin female flies. (F) Frequency of mitotic GSCs was counted by staining with anti-phospho-histone H3, which is a marker for mitotic cell division. Mated females showed an increased rate of mitotic GSCs as compared with virgin females. (G) The number of cap cells was counted by staining with anti-Lamin-C antibody, which is a marker for the cap cells. The number of germaria analyzed is shown in parentheses in E, and inside bars in D, F, and G. (H, I) Temporal change in GSC numbers in virgin and mated females. Females were mated with males for the first time 3 days after eclosion (1^st^ mating) (H) and in the second time 7 days after 1^st^ mating (2^nd^ mating) (I). Also see S1 Fig. The number of germaria analyzed for H and I are shown in S2 Table. Values are presented as the mean with standard error of the mean in G, H, I. For statistical analysis, a Mann-Whitney *U* test was used for D, H, I. Chi-square analysis was performed for F. A Student’s t-test was used for G. ***P ≤ 0.001, **P ≤ 0.01, n.s., non-significant (*P* > 0.05).

Gametes originate from a critical cell population called germline stem cells (GSCs) in most animals including *Drosophila*. *Drosophila* GSCs reside in a specialized microenvironment, or niche, where they are exposed to local signals required for stem cell function (Fig 1A) [2,3]. A number of studies have reported that the niche has an important role in regulating GSC proliferation and maintenance. However, little is known about whether and how GSC number is regulated by the signals from the external environment.

In this study, we tackle this question and show that mating stimulates GSC proliferation in female *Drosophila*. We find that mating-induced increase in GSC number is regulated by the Sex Peptide (SP) signaling pathway, a canonical pathway stimulated by seminal fluid from males. We also identify ecdysteroid as a mediator of mating-induced increase in GSC number under the control of neuronal SP signaling. Our study provides evidence that there is an essential link between neuronal SP signaling and ovarian ecdysteroid signaling to control GSC activity that may sustain reproductive output.

## Results

### Mating stimulates GSC proliferation without increased niche size

To address how gametogenesis may be stimulated upon mating, we first examined the number of female GSCs (Fig 1B) in the absence or presence of mating. Three day old females were mated with males and used for assay 1 day after mating (Fig 1C). We found that the mated female flies had significantly more GSCs compared with the virgin females (Fig 1D and S1 Table). We also found that almost all GSCs in the mated females were immunostained with an antibody against phosphorylated Mad (pMad), a critical marker for GSC identity [4], suggesting that increased GSCs after mating are functional stem cells (Fig 1E). Furthermore, mated female flies displayed an increased frequency of mitotic GSCs positive for phospho-histone H3 (Fig 1F), indicating that mating stimulates GSC proliferation as well. Mating did not increase the number of cap cells, a critical GSC niche component [2] (Fig 1G), demonstrating that mating-induced GSC proliferation is achieved without changing overall niche architecture.

We observed a significant increase in GSC numbers at 6 days, but not at 7 days after the 1^st^ mating (Fig 1H and S1A Fig and S2 Table). 7 days after the 1^st^ mating, females that were the 2^nd^ mated with males showed an increase in GSC numbers after mating again (Fig 1H and S1A Fig and S2 Table). This increase in GSC numbers was also sustained for 6 days, but not 7 days after the 2^nd^ mating (Fig 1I and S1B Fig and S2 Table). The length of this increase period is consistent with the effect of male seminal fluid as most of the sperm is lost about 1 week from first mating [5,6]. We speculated that an increase in GSC numbers after mating occurred in a seminal fluid-dependent manner.

### Neuronal sex peptide signaling regulates mating-induced increase in GSC numbers

**Fig 2:**
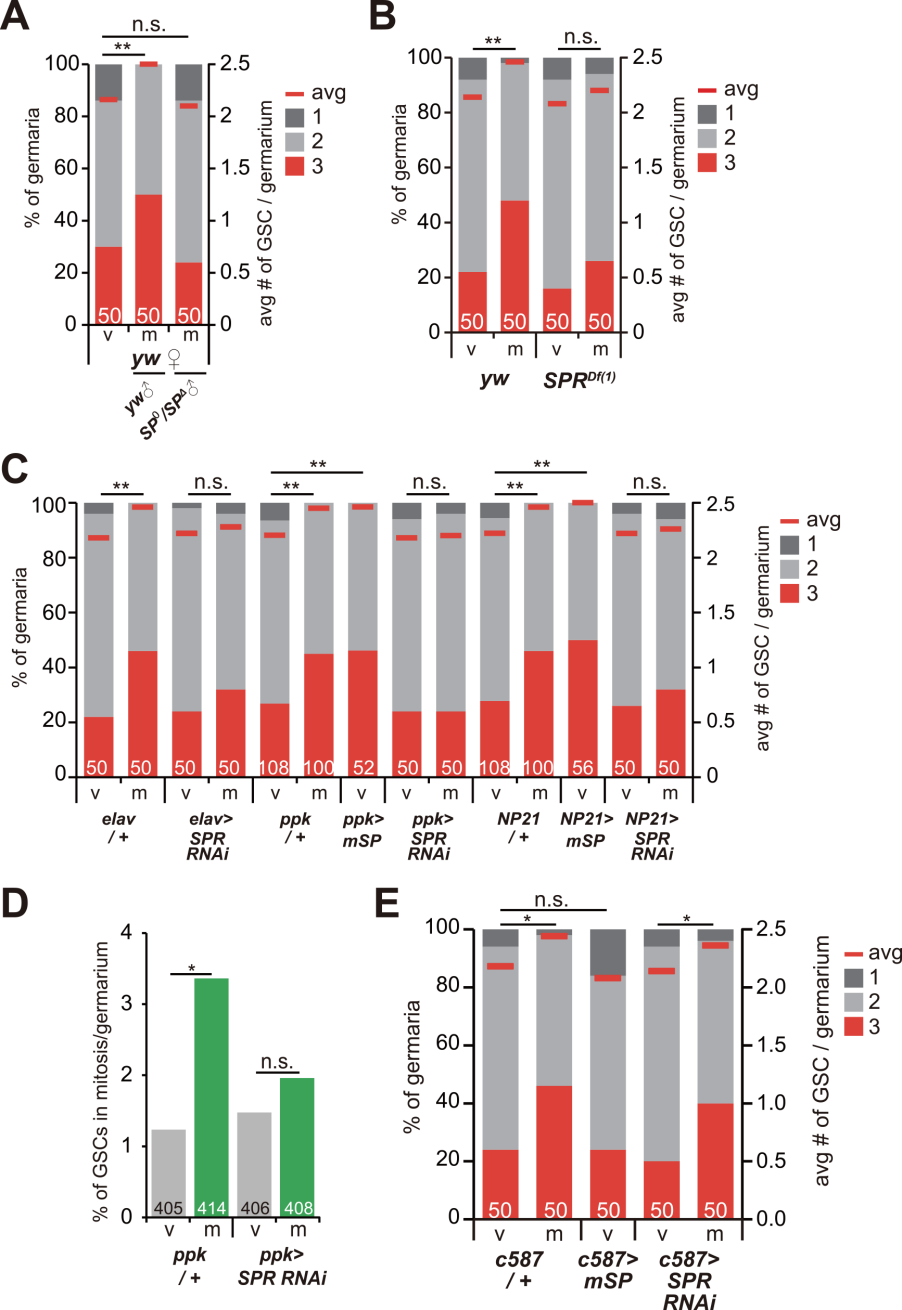
Neuronal *SPR* function is required and sufficient for a mating-induced increase in GSC numbers. (A, B, C and E) Frequencies of germaria containing one, two, and three GSCs (left y axis), and average number of GSCs per germarium (right y axis) in virgin (v) and mated (m) female flies. (A) Wild-type females were mated with wild-type and *SP* trans-heterozygous mutant adult male flies (*SP*^*0*^/*SP*^*Δ*41^). (B) Wild-type or *SPR* null homozygous female flies (*SPR*^*Df(1)Exel6234*^) were mated with wild-type male flies. (C, E) Adult female flies overexpressing the membrane bound form of *SP* (*mSP*) or transgenic *SPR* RNAi were mated with wild-type male flies under the control of several neuronal GAL drivers (*elav-GAL4*, *ppk-GAL4* and *fru (NP21)-GAL4*) (C) or ovarian somatic cell-specific GAL4 driver (*c587-GAL4*) (E). Transgenes were driven by indicated GAL4 drivers. *NP21-GAL4* was used for driving transgenes in *fru*-positive neurons. (D) Frequency of mitotic GSCs was counted by staining with anti-phospho-histone H3 in *SPR* RNAi female flies. The numbers of germaria analyzed are shown inside the bars. For statistical analysis, a Mann-Whitney *U* test was used for (A, B, C and E). Chi-square analysis was performed for (D). **P ≤ 0.01, *P ≤ 0.05, n.s., non-significant (*P* > 0.05).

Several behaviors specific to post-mated female flies, such as oviposition of eggs and a rejection of courtship with male flies, are induced by a peptide in male seminal fluid (SP) [7]. We therefore examined whether SP signaling is involved in the mating-induced increase in GSC numbers. We found that wild-type female flies did not show an increase in GSC numbers after mating with *SP* null (*SP*^*0*^) male flies (Fig 2A and S1 Table).

SP is received by female neurons expressing the specific receptor gene *sex peptide receptor* (*SPR*) [8,9]. Consistent with this finding, we found that loss of *SPR* function in female flies did not cause a mating-induced increase in GSC numbers even after mating with wild-type males (Fig 2B and S1 Table). The *SPR*-positive neurons located on the oviduct, which also co-express *pickpocket* (*ppk*) and *fruitless* (*fru*), are particularly crucial in inducing the major behavioral changes in female flies after mating [10]. We therefore examined the number of GSC phenotypes in transgenic RNAi animals knocking down *SPR* function in *ppk*-positive neurons, *fru*-positive neurons and almost all post-mitotic neurons using *ppk-GAL4* [11], *NP21-GAL4* [12,13] and *elav-GAL4* [14] drivers, respectively. Similar to the observation with the genetic *SPR* mutant animals, all of these neuronal *SPR* RNAi animals did not exhibit a mating-induced increase in GSC numbers in mated females (Fig 2C and S1 Table). In addition, the neuronal *SPR* RNAi disrupted an increase in mitotic GSCs after mating (Fig 2D), suggesting that SP signaling is required for mating-induced GSC proliferation. Conversely, we also found that a forced expression of the *SP* gene in either *ppk*- or *fru*-positive female neurons increased GSC numbers even in virgin female flies (Fig 2C and S1 Table). On the other hand, this GSC increase in virgin female flies was not observed by overexpression of *SP* in ovarian somatic cells using *c587-GAL4* driver (Fig 2E and S1 Table). In addition, the mating-induced increase in GSCs still occurred in the ovarian *SPR* RNAi animals (Fig 2E and S1 Table). These results suggest that neuronal, but not ovarian, SP signaling is both necessary and sufficient for an increase in GSC numbers.

SP is sufficient to increase egg laying in virgin females [15,16]. To distinguish the impact of SP signaling versus egg laying on an increase in GSC numbers, we measured GSC numbers in virgin females that lay many eggs without SP signaling. As previously reported [17], we confirmed that virgin females laid many eggs, as many as mated females did when they were fed yeast paste, a protein source that promotes egg production (S2A Fig). Nevertheless, we found that virgin females that laid many eggs did not show a significant increase in GSC numbers (S2B Fig and S1 Table). These results suggest that an increase in GSC numbers requires SP signaling but not stimulation of unfertilized egg laying in virgin females.

### Mating-induced ovarian ecdysteroid biosynthesis mediates an increase in GSC number after mating

**Fig 3:**
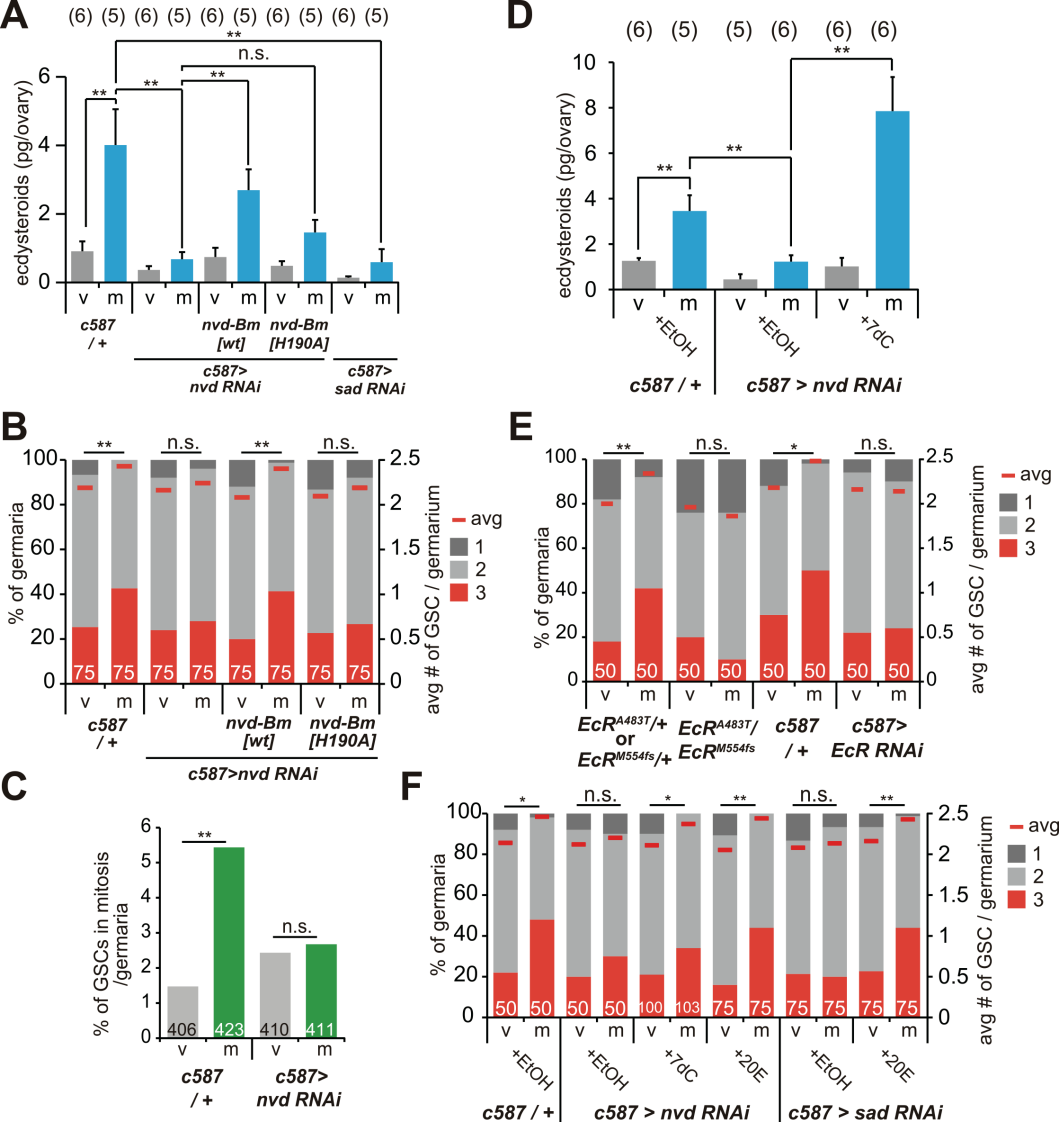
Ovarian ecdysteroid biosynthesis controls a mating-induced increase in GSC numbers. (A, D) Ecdysteroid levels in virgin (v) and mated (m) females in the ovarian somatic cell-specific (escort cells and follicle cells) *nvd* RNAi female flies. (A) *c587-GAL4* driver was crossed with control or *UAS* transgene strains as indicated. *UAS-nvd-Bm [wt]* and *UAS-nvd-Bm [H190A]* overexpressed the wild-type form and enzymatic inactive form of *Bombyx mori nvd* transgenes, respectively. (D) *nvd* RNAi female flies were fed food supplemented with ethanol (EtOH; for control) and 7-dehydrocholesterol (7dC). (B, E and F) Frequencies of germaria containing one, two, and three GSCs (left y axis), and average number of GSCs per germarium (right y axis) of follicle cell-specific *nvd* RNAi animals with or without the *B*. *mori nvd* transgene (B), the ovarian somatic cell-specific *EcR* RNAi female flies and transheterozygous mutants for *EcR* (*EcR*^*A483T*^ and *EcR*^*M554fs*^, mutants in the predicted ligand- binding domain) (E), ovarian somatic cell-specific *nvd* and *sad* RNAi female flies that were fed food supplemented with EtOH (for control), 7dC and 20E (F). (C) Frequency of mitotic GSCs was counted by staining with anti-phospho-histone H3 in *nvd* RNAi female flies. Values are represented as the mean with standard error of the mean in A and B. The numbers of samples examined are indicated in parentheses in A and D. The numbers of germaria analyzed are shown inside bars in B, C, E and F. For statistical analysis, Dunnett’s test was used for A and D. A Mann-Whitney *U* test was used for B, E and F. Chi-square analysis was used for C. **P ≤ 0.01, *P ≤ 0.05, n.s., non-significant (*P* > 0.05).

To explore which signaling mechanism(s) might mediate a mating-induced increase in GSC numbers, we examined the principal insect steroid hormone, ecdysteroid. The ecdysteroid signaling in the ovary is involved in the long-lasting maintenance of GSC numbers throughout adult life [18–22]. A previous study has reported that the ecdysteroid titer in the female’s whole body increases after mating, assuming that ovarian ecdysteroid activated by mating [23]. However, it has never been experimentally tested whether and how ovarian ecdysteroid biosynthesis is affected by mating, or is involved in an increase in GSC numbers. To address this, we measured the ecdysteroid level in virgin and mated female ovaries and found that mating increased the ecdysteroid level in the ovaries of mated female flies as compared with virgin female flies (Fig 3A). The increase in the ovarian ecdysteroid level was not simply owing to an increase in ovarian mass, since weights of ovaries derived from virgins were heavier than from mated female flies (S3 Fig). To examine whether a mating-induced increase in the ecdysteroid level is owing to *de novo* biosynthesis in the ovary, we generated adult ovary-specific RNAi animals for *neverland* (*nvd*), which encodes the ecdysteroidogenic enzyme responsible for converting dietary cholesterol into 7-dehydrocholesterol (7dC) [24,25]. We knocked down *nvd* expression with *c587-GAL4* driver that was active in the ovarian somatic cells (escort cells and undifferentiated follicle cells). The ovarian somatic cell-specific *nvd* RNAi females displayed reduced Nvd protein levels in the follicle cells (S4A Fig). We confirmed that the *nvd* RNAi mated female flies did not exhibit an increase in ovarian ecdysteroid level after mating, but exhibited a significant reduction when compared with the genetic controls (*c587-GAL4/+*) (Fig 3A). Impairment of the mating-induced ovarian ecdysteroid biosynthesis was restored by co-expression of the wild-type form, but not the enzyme-dead form, of the silkworm *Bombyx mori* orthologue of *nvd* (*nvd-Bm*) (Fig 3A), suggesting that the ovarian *nvd* RNAi phenotype is not an off-target effect.

In the ovarian *nvd* RNAi animals, we observed that neither the total number of GSCs (Fig 3B and S1 Table) nor the number of mitotic GSCs (Fig 3C) increased after mating. Consistent with the ecdysteroid level data, the suppression of a mating-induced increase in GSC numbers in the *nvd* RNAi animals was rescued by co-expression of the wild-type form of *nvd-Bm*, but not the enzyme-dead form in mated females (Fig 3B and S1 Table). We also confirmed that the low ecdysteroid level phenotype in the *nvd* RNAi mated female flies was rescued by oral administration of 7-dehydrocholesterol, the downstream metabolite generated by Nvd (Fig 3D). The *nvd* RNAi females fed 7dC showed a significant increase in GSC numbers in mated females as compared with virgin females (Fig 3F and S1 Table). The phenotype of GSC number in *nvd* RNAi was also rescued by oral administration of 20-hydroxyecdysone (20E), the biologically active ecdysteroid (Fig 3F and S1 Table). To further examine the role of mating-induced ecdysteroid biosynthesis in controlling ovarian GSC numbers, we conducted an additional experiment using the ovary-specific RNAi for *shadow* (*sad*), which is another ecdysteroid biosynthesis enzyme [26]. The ovarian *sad* RNAi reduced the Sad protein level in the follicle cells (S4B Fig) and exhibited the same phenotypes as observed using the ovarian *nvd* RNAi female flies (Fig 3A and 3F and S1 Table).

Previous studies have shown that *nvd* and *sad* transcripts are expressed not only in the follicle cells but also in the nurse cells [24,26]. However, knocking down *nvd* or *sad* expression in the nurse cells using *nanos* (*nos*)*-GAL4* had no effect on the mating-induced increase in GSCs (S5 Fig and S1 Table). We conclude that ecdysteroids biosynthesized in the follicle cells are essential for inducing a mating-induced increase in GSC numbers.

### Mating-induced ovarian ecdysteroid increases GSC numbers via *Ecdysone receptor* expressed in the ovary

To examine whether ecdysteroids are received in the ovary, we used flies with loss of *ecdysone receptor* (*EcR*) function, which encodes a receptor component for 20E. We found that a mating-induced increase in GSC numbers was suppressed in either hypomorphic alleles or *c587-GAL4*-driven RNAi of the *EcR* gene (Fig 2E and S1 Table). Although it has been shown that ecdysteroids directly act on GSCs to control long-lasting GSC maintenance in aged females [18], we found that knock down of *EcR* function in germline cells including GSCs did not affect mating-induced increase in GSCs (S5 Fig and S1 Table). This result suggests that the ecdysteroid signaling controls mating-induced increase in GSCs via different mechanisms that regulate long-lasting GSC maintenance during aging. Taken together, these data suggest that ecdysteroid biosynthesis in the ovary is activated by mating stimuli, and the ovarian ecdysteroid signaling mediates a mating-induced increase in GSC numbers.

### Ovarian ecdysteroid biosynthesis is required for reproductive output

**Fig 4:**
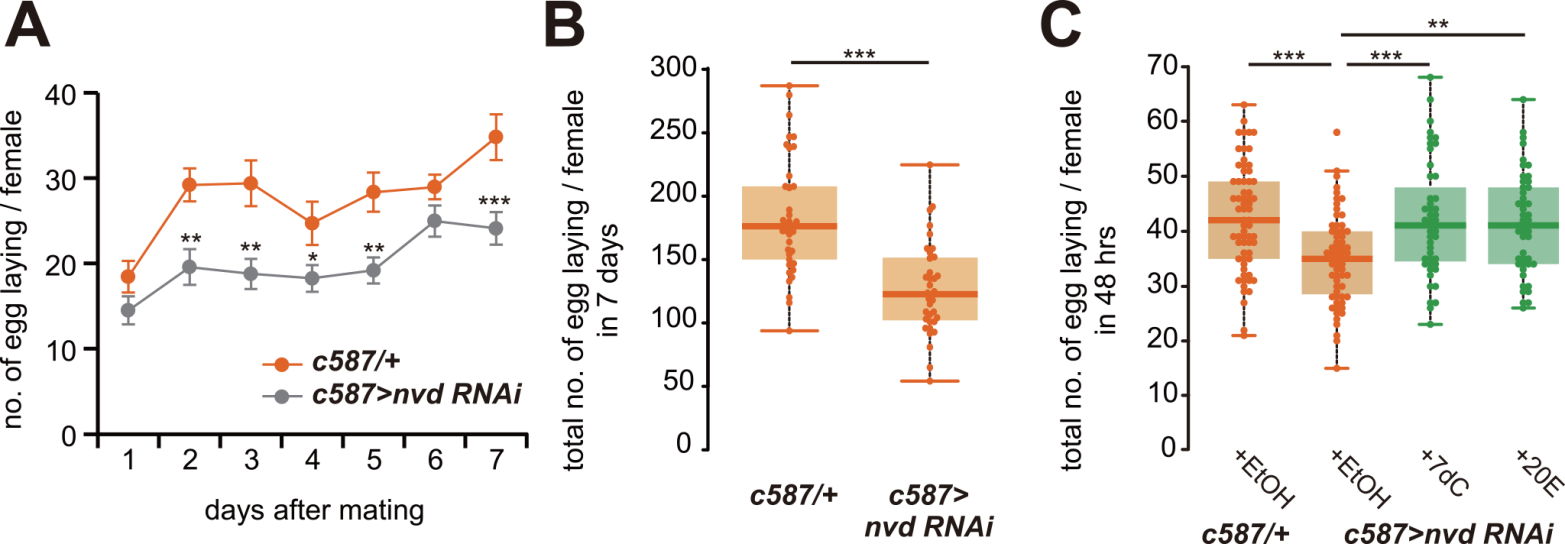
Ovarian ecdysteroid biosynthesis is required for reproductive output. (A–C) The number of laid eggs was measured in ovaries of follicle cell-specific *nvd* RNAi female flies. (A) Temporal changes in laid eggs were measured for 7 days. (B) Total number of laid eggs in 7 days after mating is shown. (C) Total number of laid eggs in 48 hours in *nvd* RNAi female flies that were fed food supplemented with EtOH (for control), 7dC and 20E. Feeding of 7dC or 20E rescued decreased egg number phenotype in *nvd* RNAi. Values are presented as the mean with standard error of the mean in A. Each value is plotted as a dot. Box plot shows 25–75% (box), median (band inside) and minima to maxima (whiskers) in B and C. For statistical analysis, a Student’s t-test was used for A and B. Dunnett’s test was used for C. ***P ≤ 0.001, **P ≤ 0.01, *P ≤ 0.05.

To test if the mating-induced increase in the ovarian ecdysteroid levels is required for reproductive output, we performed an egg laying assay to count the number of laid eggs from the females of the ovarian somatic cell-specific *nvd* RNAi females. We found that the *nvd* RNAi females showed a significant reduction in the number of laid eggs as compared with the control females (Fig 4A and 4B). We also checked that the phenotype of the number of laid eggs in the *nvd* RNAi was restored by oral administration of 7dC or 20E (Fig 4C). Taken together, these data suggest that ovarian ecdysteroid biosynthesis after mating is required for both an increase in egg and GSC numbers.

### The insulin signaling pathway is not required for a mating-induced increase in GSC numbers

In addition to ecdysteroid, the insulin signaling pathway plays an important role in controlling age- and diet-dependent GSC maintenance in females [27–29]. To examine whether insulin signaling induces a mating-induced increase in GSC numbers, we measured GSC numbers in flies expressing *InR RNAi* and a dominant-negative form of *InR in* follicle cells. A significant increase in GSC numbers still occurred after mating in the loss of the *insulin receptor* gene function of flies (S6A Fig and S1 Table), whereas female flies with the same genotypes showed age-dependent GSC loss as previously reported (S6B Fig and S1 Table) [27–29]. Consistent with this data, mating-induced GSC increase still occurred in female flies fed protein-free diet for 2 days whereas these flies had smaller ovaries than control’s one (S6C Fig and S1 Table). These results suggest that the insulin signaling pathway is not involved in controlling a mating-induced increase in GSC numbers and mating-induced GSC proliferation is controlled by the mechanisms distinct from age- and diet-dependent GSC maintenance.

### The juvenile hormone signaling pathway is not required for a mating-induced increase in GSC numbers

Juvenile hormone (JH) is another important insect hormone, which controls oocyte maturation [30]. We examined a role of JH signaling in regulating the mating-induced increase in GSC numbers. While an application of methoprene, the juvenile hormone analogue (JHa), mimics the mating-induced stimulation of vitellogenic oocyte progression in virgin females [30], JHa feeding had no effect on the number of GSCs in virgins and mated female flies (S7A Fig and S1 Table). In addition, mating-induced GSC increase still occurred in the female flies whose JH production was blocked by overexpression of the protein phosphatase inhibitor *NiPp1* [31,32] or transgenic RNAi for *juvenile hormone acid O-methyltransferase* (*JHAMT*) [33] in the corpora allata using *Aug21-GAL4* driver (S7B Fig and S1 Table). Consistent with this observation, we observed a significant increase in GSC numbers after mating in RNAi flies for JH receptors *Methoprene-tolerant (Met)* or *germ cell-expressed bHLH-PAS (gce)*, or their target *Kr-h1* [34] in the ovarian somatic cells the same way as control flies (S7C Fig and S1 Table). Taken together, we conclude that JH signaling is not required for mating-induced GSC increase.

### SP signaling positively controls ecdysteroid biosynthesis to induce mating-induced increase in GSC numbers

**Fig 5:**
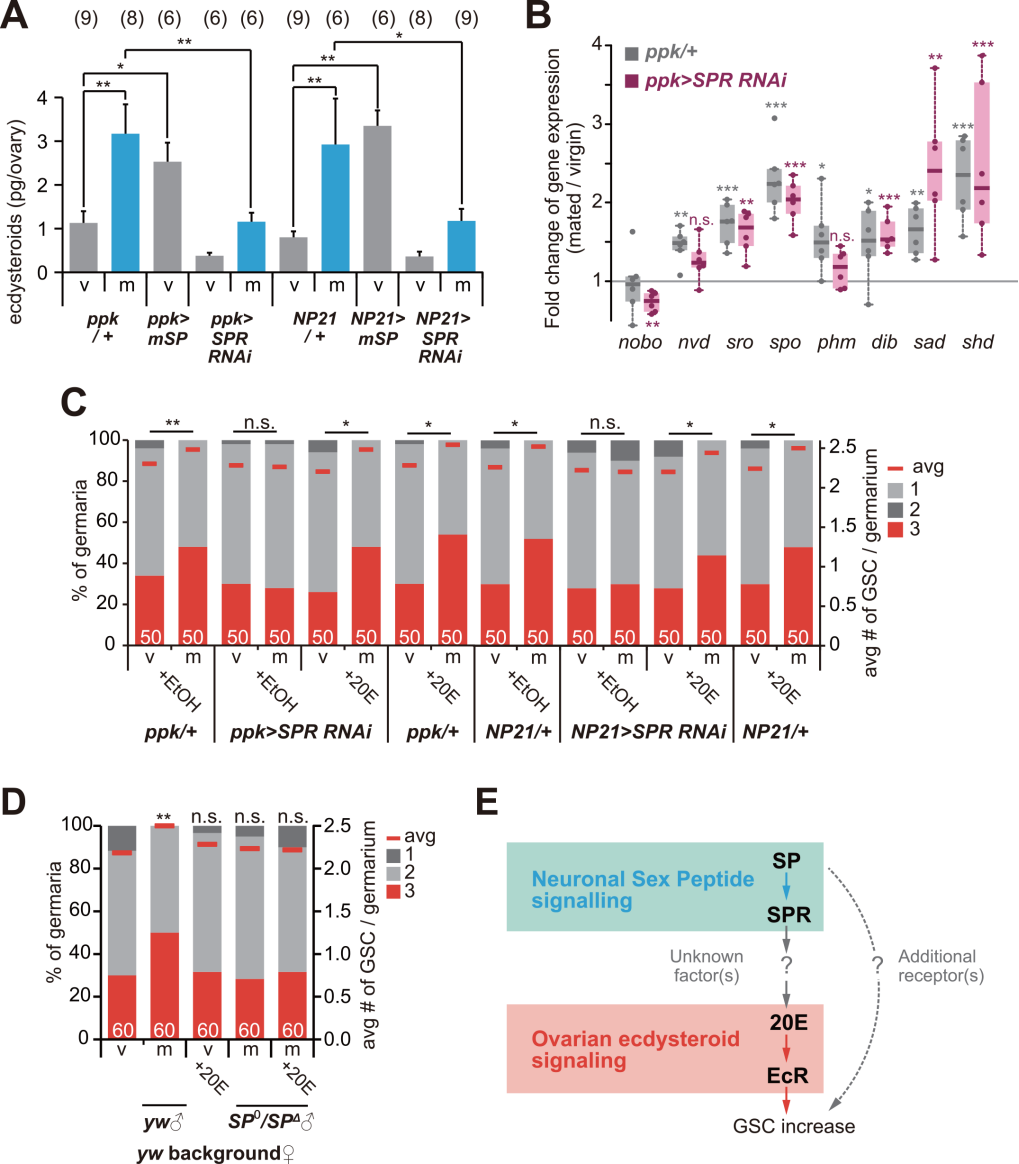
Neuronal SP signaling controls a mating-induced increase in GSC numbers dependent on ecdysteroid. (A) Ecdysteroid levels in virgin (v) and mated (m) female ovaries isolated from adult female flies overexpressing the membrane bound form of *SP* (*mSP*) and transgenic *SPR* RNAi. Transgenes were driven by *ppk-GAL4* or *fru (NP21)-GAL4*. (B) Mating-induced expression of ecdysteroid biosynthesis enzyme genes in the ovaries. The *y*-axis represents fold changes of transcript levels between the ovaries of mated and virgin females. Box plot shows 25–75% (box), median (band inside) and minima to maxima (whiskers). Most of the enzyme genes, except for *nobo*, transcriptionally increased by mating in control female ovaries (Gray: *ppk/+*). Increased transcript levels of *nvd* and *phm* by mating were suppressed in *SPR* RNAi females (Purple: *ppk>SPR RNAi*). (C and D) Frequencies of germaria containing one, two, and three GSCs (left y axis), and average number of GSCs per germarium (right y axis) in virgin (v) and mated (m) female flies of neuronal *SPR* RNAi adult female flies that were fed food supplemented with EtOH (for control) and 20E. (D) Females mated with *SP* null mutant males did not show mating-induced increase in GSCs and this phenotype was not rescued by oral administration of 20E. (E) Schematic of neuroendocrine control of a mating-induced increase in GSC numbers. Neuronal sex peptide signaling induced by mating increases GSC numbers via activated ovarian ecdysteroid biosynthesis. Since overexpression of *SP*, but not feeding of 20E into virgin females was sufficient to induce an increase in GSC numbers, there might be another pathway activated by *SP* to control GSC numbers (dotted arrow). Values are presented as the mean with standard error of the mean in A. The numbers of samples examined are indicated in parentheses in A, or inside bars in C, D. For statistical analysis, Dunnett’s test was used for A, Student’s t-test was used for B, a Mann-Whitney *U* test were used for C and D. ***P≤ 0.001, **P ≤ 0.01, *P ≤ 0.05, n.s., non-significant (*P* > 0.05).

Finally, we addressed whether SP signaling regulates the mating-induced increase in GSC numbers via ecdysteroid. We found that a mating-induced increase in the ovarian ecdysteroid level was significantly suppressed in the mated female flies of neuronal *SPR* RNAi (Fig 5A). We also found that the ovarian ecdysteroid level increased in the neuronal forced expression of *SP* in virgin female flies (Fig 5A), suggesting that SP signaling is both necessary and sufficient for activating ovarian ecdysteroid biosynthesis.

We also examined whether mating and SP-SPR signaling affect transcription levels of ecdysteroid biosynthesis enzyme genes, as the enzyme genes are transcriptionally regulated by several humoral factors during development [35]. The expression levels of most of ecdysteroidogenic enzyme genes (*nvd*, *sro*, *spo*, *phm*, *dib*, *sad* and *shd*) in the mated female ovaries were up-regulated after mating (Fig 5B, gray bars). Moreover, the transcriptional increase of *nvd* and *phm* were significantly impaired in neuronal *SPR* RNAi adult female flies (Fig 5B, purple bars), suggesting that mating-dependent transcriptional up-regulation of ecdysteroidogenic enzyme genes is partially, but not fully, regulated by SP-SPR signaling.

Importantly, the GSC number phenotype of the *ppk-GAL4*- or *NP21-GAL4*-driven *SPR* RNAi mated female flies is owing to ecdysteroid loss, because the GSC phenotype was rescued by oral administration of 20E (Fig 5C and S1 Table). In contrast, curiously, the impairment of GSC increase in female flies mated with *SP* null males was not rescued by oral administration of 20E (Fig 5D and S1 Table). Taken together, our data strongly suggest that the mating-induced increase in female GSC numbers is regulated by ovarian ecdysteroid whose biosynthesis is positively controlled by neuronal SP signaling in mated female flies, while there must be an unknown pathway dependent on SP but independent of the SPR-ecdysteroid cascade (Fig 5E).

## Discussion

As far as we know, this is the first study to show that GSC proliferation is under the control of the characterized neuroendocrine system in response to mating, an external stimulus. In many insects, mating triggers dramatic changes in female behaviors, such as reduced receptivity and increased egg laying, which enhance reproductive output [1,7]. In *Drosophila*, the behavioral shift, known as the post-mating response, is activated by the seminal peptide SP via binding to SPR that causes silencing of *SPR*-positive neurons [9,36]. Although *SPR*-positive neurons are detected in the female reproductive tract, ventral nerve chord, and brain [8,37], a restricted subset of *SPR*-positive neurons located on the reproductive tract, which is also *ppk*- and *fru*-positive, is necessary and sufficient for inducing many aspects of the post-mating response [8,10]. We here propose that SP-SPR signaling in the shared reproductive tract neurons also play a crucial role in regulating GSC proliferation (Fig 5E). Previous study has reported that *SPR*-positive neurons on the reproductive tract innervate the downstream SP abdominal ganglion neurons, which further project and signal into the central brain to modulate post-mating switch [36]. It is interesting to examine how shared these downstream neurons are to control mating-induced GSC proliferation and the canonical post-mating response, whereas the precise neuronal circuit transmitting the SP signal has not yet fully elucidated.

Our results show that ovarian ecdysteroids are required for post-mating increase in GSC numbers. Previous studies have shown that EcR and the downstream signaling molecules are required for maintaining female GSCs during the aging process in *Drosophila* without a change of cap cell numbers [18–20]. Our study, in conjunction with these previous results, suggests that ecdysteroid signaling is essential for not only long-lasting maintenance, but also the mating-induced acute proliferation.

In contrast to the necessity of the ecdysteroid signaling for GSC proliferation, our results suggest that ecdysteroids appear not to be sufficient for the phenomena, as oral 20E administration itself does not increase GSC numbers in virgin female flies (Figs 3F and 5C). Thus, the ovarian ecdysteroid appears to be a permissive, but not instructive, factor for mating-induced increase in GSCs. Related to the insufficiency of ecdysteroids, our data also suggest that SP-dependent induction of GSC increase appears not to be solely due to the SPR-ecdysteroid cascade (Fig 5E). First, *SP* overexpression is sufficient to induce ovarian ecdysteroid biosynthesis (Fig 5A) and GSC proliferation (Fig 2C). Second, the GSC phenotype in *ppk/fru*-promoter-driven *SPR* RNAi animals is rescued by oral administration of 20E (Fig 5C). Third, nevertheless, the impairment of GSC increase in female flies mated with *SP* null males is not rescued by oral administration of 20E (Fig 5D and S1 Table). These results imply that there must be an unknown pathway that acts downstream of SP and is independent of the SPR-ecdysteroid cascade (Fig 5E). In facts, a recent study has proposed that there must be additional receptor(s) required for inducing SP-dependent post-mating switch [38]. Elucidating such additional receptor(s) and its/their signaling pathway would be important to comprehensively understand the mating-induced GSC proliferation in future.

Our data strongly suggest that mating-induced ecdysteroid biosynthesis in the ovary is stimulated in a neuronal SP signaling-dependent manner. It is worth to mention previous studies showing that SP also induces the production of another indispensable insect hormone JH by stimulating the corpus allatum [39,40]. More recently, it has been reported that JH controls mating-induced increase in fecundity in *Drosophila* [32]. In this case, mating activates JH biosynthesis in the corpora allata, and the increase in JH titer induces intestine remodelling, resulting in an increase in midgut size and cell number [32]. However, in our case, application of JHa and blocking JH signaling do not affect the mating-induced GSC increase (S6 Fig), suggesting that JH acts on the ovary to regulate oocyte maturation but not GSC increase. Such 20E-dependent and JH-independent manner of mating-induced GSC increase is contrast to the mechanism regulating oocyte maturation, in which there is the hormonal crosstalk between 20E and JH; JH stimulates vitellogenesis through a control point at about stage 9 of oogenesis while 20E induces apoptosis in nurse cells of early vitellogenic egg chambers [7].

The important question to be addressed in the future is which factor(s) directly stimulate ovarian ecdysteroid biosynthesis downstream of SP signaling. In the larval stages, several peptidergic and monoaminergic factors play pivotal roles in stimulating ecdysteroid biosynthesis in the endocrine organ called the prothoracic gland [41–43]. However, molecular mechanisms regulating ovarian ecdysteroid biosynthesis are still poorly understood in *Drosophila*. It can be assumed that *SPR* is expressed in ovarian somatic cells, and SP might be directly received by SPR in the ovaries, as SPR suppresses ecdysteroid biosynthesis by receiving a neuropeptide during larval development of the silkworm *Bombyx mori* [44]. However, based on immunohistochemistry using anti-SPR antibody, it is unlikely that SPR is present in the ovary [8]. Consistent with this observation, we show that *SPR* knockdown or *SP* expression by neuronal GAL4 driver, but not the ovary-specific GAL4 driver, affect mating-induced increase in GSC numbers (Fig 2E). These data imply an existence of indispensable neuronal and/or humoral factors that connect SP-SPR signaling in the reproductive tract neurons with ovarian ecdysteroid biosynthesis for mating-triggered GSC increase.

A possible candidate of such a factor is *Drosophila* insulin-like peptides (DILPs), as neural-derived DILPs are the crucial systemic factors in controlling GSC proliferation in response to diet and age [28,29]. In addition, in the mosquito *Aedes aegypti*, ILPs stimulate ovarian ecdysteroid biosynthesis [45,46]. However, it is unlikely that the mating-induced increase in GSC numbers requires the DILP pathway. First, our data indicate that with a loss of *InR* function, females exhibit an increase in GSC numbers after mating (S5A Fig). Second, it has been reported that neuronal DILPs control GSC maintenance through regulation of the number of cap cells, which constitute a crucial cell population for niche [29]. It should be noted that mating stimulus does not evoke such an increase in cap cell number (Fig 1G). These data suggest that a mating-induced increase in GSC numbers is regulated by a pathway that is different from the neuronal DILP signaling pathway required for long-lasting GSC maintenance. Besides ILPs, in the mosquito *A*. *aegypti*, the neuropeptide called ovary ecdysteroidogenic hormone (OEH) is also known to induce ovarian ecdysteroid biosynthesis and regulate egg maturation [47,48], while a role of OEH in GSC proliferation has not yet been examined. Somehow it seems that orthologues of *OEH* genes have been lost from the *D*. *melanogaster* subgroup [48,49]. It would therefore be intriguing to identify and characterize specific humoral factor(s) controlling ovarian ecdysteroid biosynthesis in response to SP signaling in *D*. *melanogaster*.

It is widely accepted that proliferation and maintenance of stem cells are affected by internal signals and external environments in many animals including some mammals [50]. Because a mating stimulus is known to influence many aspects of female physiology and behavior not only in insects, but also in mammals [51], it would be intriguing to investigate whether mating also affects GSCs in higher animals and, if so, which neuronal and endocrine systems are involved in this phenomena.

## Materials and Methods

### Fly strains and transgene rescue experiments

Flies were raised on cornmeal-agar-yeast media at 25°C. *yw* was used as the control strain. *SPR*^*Df(1)Exel6234*^ generated by Exelixis, Inc.[8] was obtained from the Bloomington Drosophila Stock Center (BDSC, stock number #7708). *w;dj-GFP/CyO* (#5417) [52], *UAS-InR-IR* (#31039), *UAS-InR-DN* (#8251), *elav-GAL4* (#8765) [14], *UAS-NiPp1* (#23711) [31], *EcR*^*M554fs*^ (#4894) and *EcR*^*A483T*^ (#5799) [53] were also obtained from BDSC. *nos-GAL4* (#107748) was obtained from the Drosophila Genetic Resource Center at Kyoto. *UAS-SPR-IR* (VDRC KK106804), *UAS-sad-IR* (VDRC KK106356), *UAS-EcR-IR* (VDRC KK37059), *UAS-Met-IR* (VDRC KK100638), *UAS-gce-IR* (VDRC KK101814) and *UAS-Kr-h1-IR* (VDRC KK107935) were obtained from the Vienna Drosophila RNAi Center. *SP*^*0*^, *SP*^*Δ*^ [54] and *NP21-GAL4*, an enhancer trap line inserted near the *fruitless* promoter [12,13], were gifts from Nobuaki Tanaka (Hokkaido University, Japan). *c587-GAL4* (ref. [55,56]), *ppk-GAL4* (ref. [11]) and *Aug21-GAL4* (ref. [57]) were gifts from Hiroko Sano (Kurume University, Japan), Tadashi Uemura (Kyoto University, Japan) and Günter Korge, respectively. *UAS-SP*.*GGT* for overexpressing a transgene of membrane-bound form of *SP* [16] was a gift from Toshiro Aigaki (Tokyo Metropolitan University). Other strains used were *UAS-nvd-IR*, *UAS-nvd-Bm [wt]*, *UAS-nvd-Bm [H190A]* (ref. [25]).

### Mating assay

Flies were reared at 25°C and aged for 3 days. About 15–30 virgin female flies were introduced into a vial with cornmeal-agar-yeast media containing more *yw* male flies than female flies for 6 h at 25°C. The mating protocol using 3- or 4-day-old adult female flies has also been used in previous studies to assess mating-induced *Drosophila* behaviors. Individual female flies were introduced into a vial with yeast paste on grape juice-agar media for 16 h (for GSC count) or 24 h (for ecdysteroid measurement) at 25°C. We determined mating success by checking egg laying and hatching larvae. For inducing egg laying in virgin females, we put virgin females on yeast paste on a grape plate for 48 h as previously described [17]. We transferred flies into fresh vials and counted egg-number manually after 48 h.

### Immunohistochemistry

Ovaries were dissected in Grace’s Insect Medium, Supplemented (Life Technologies) and fixed in 4% paraformaldehyde in Grace’s medium for 20 min at room temperature (RT). For staining of germaria, the fixed samples were washed three times in phosphate-buffered saline (PBS) supplemented with 0.1% Triton X-100. For staining of whole ovarioles, the fixed samples were washed twice in PBS with 1% Triton X-100 and once in PBS with 2% Triton X-100. After these washing steps, the samples were blocked in the blocking solution (PBS with 0.3% Triton X-100 and 0.2% BSA) for 1 h at RT, and then incubated with a primary antibody in the blocking solution at 4°C overnight. The primary antibodies used in this study were mouse anti-Hts 1B1 (ref. [58]) (1:50; purchased from Developmental Studies Hybridoma Bank), rat anti-*D*E-cadherin DCAD2 (ref. [59]) (1:100; purchased from Developmental Studies Hybridoma Bank), rabbit anti-phospho-histone H3 (Millipore; 1:1000), rabbit monoclonal anti-pMad (Abcam; 1:1000), mouse anti-Lamin-C LC28.26 (ref. [60])(1:10; provided from Satoru Kobayashi, National Institute for Basic Biology, Japan), rabbit anti-Sad [61] (1:200; a gift from Michael B. O’Connor, University of Minnesota, USA), and guinea pig anti-Nvd [43] (1:200). When anti-pMad antibody was used, the immunostaining signals were enhanced by Can Get Signal Solution B (Toyobo). Fluorescent (Alexa488 or Alexa546)-conjugated secondary antibodies (Life Technologies) were used at a 1:200 dilution and incubated for 2 h at room temperature in the blocking solution. All samples were mounted in FluorSave reagent (Calbiochem). GSC numbers were determined based on the morphology and position of their anteriorly anchored spherical spectrosome.

### Ecdysteroid level measurements

Flies were reared at 25°C and aged for 4 days. Ovaries were dissected in PBS. Samples were homogenized in 50 μL of methanol in 1.5-mL tubes using a pestle. After centrifugation at 20,913 x g for 1 min, the supernatants were transferred to new tubes and dried with a centrifugal evaporator. Samples were resuspended in 50 μL of EIA buffer (Cayman Chemicals) according to the manufacturer’s protocol and incubated at 4°C overnight. Ecdysteroid levels were quantified by an enzyme-linked immunosorbent assay using anti-20E antiserum (Cayman Chemicals) and 20E-acetylcholinesterase (Cayman Chemicals) as essentially described [62]. Note that the antiserum used in this study is known to recognize not only 20-hydroxyecdysone (20E) but also ecdysone [63]. In this study, we used we used 20E (ENZO Life Sciences) as a standard, and the ecdysteroid amount was expressed in 20E equivalents. Absorbance was measured at 415 nm using a microplate reader Multiskan GO (Thermo Fisher Scientific).

### Feeding experiments using 20-hydroxyecdysone, 7-dehydrocholesterol and methoprene (JH analogue)

Flies were reared at 25°C and aged for 1 day and placed on cornmeal-agar-yeast media mixed with a solution of 20E in ethanol, resulting in a final concentrations of 10^−5^ M for 3 days. Flies were also fed on regular food containing 0.1% wet weight 7-dehydrocholesterol (7dC, Sigma) or 1.5 mM methoprene (Earth Biochemical). The experiment using 7dC was carried out under constant dark conditions because 7dC is unstable in light. The same concentration of methoprene was used in a previous study for *Drosophila* female adults [32]. GSC counts or ecdysteroid measurements were performed on mated and unmated 4-day-old female flies.

### Egg laying assay

3-day-old females were transferred to a vial with the same number of males to allow mating. Individual females were transferred to a fresh vial and allowed to lay eggs for 24 hours. We used a round chamber (23 mm diameter and 40 mm height) with cornmeal-yeast-agar media.

### Quantitative reverse transcription (qRT)-PCR

To quantify mating-induced changes in gene expression, the ovaries from 2 adult female flies (4-day-old virgin or mated females) were dissected. Total RNA was extracted using RNAiso Plus reagent (TaKaRa). cDNAs were prepared with ReverTra Ace qPCR RT Master Mix with gDNA remover (ToYoBo). qRT-PCR was performed using the Universal SYBR Select Master Mix (Applied Biosystems) with a Thermal Cycler Dice TP800 system (TaKaRa). Serial dilutions of a plasmid containing the ORF of each gene were used as a standard. The amount of target RNA was normalized to an endogenous control *ribosomal protein 49* (*rp49*), and the relative fold change was calculated. The primers for quantifying *nobo*, *nvd*, *sro*, *spo*, *phm*, *dib*, *sad* and *shd* were used in the previous studies [64–66].

### Statistical analysis and graphing

Statistical analysis and graphing were performed with Excel (Microsoft) and an add-in software for statistics (Excel Toukei 2011; Social Survey Research Information). The mean values were calculated with standard errors. For analyzing the values of GSC numbers, a Mann-Whitney *U* test was applied. For analyzing the values of number of cap cells, number of laid eggs, ecdysteroid levels, and weight of ovaries, Student’s *t*-test or Dunnett’s test (For multiple comparisons) were applied. For analyzing the values of frequency of mitotic GSCs, Chi–square analysis was applied. The *P* value is provided for comparison with the control shown as *P ≤ 0.05, **P ≤ 0.01, ***P ≤ 0.001; n.s., non-significant (*P* > 0.05).

## Supporting Information

S1 Table Source data for the number of germaria containing one, two or three GSCs represented in the main figures. Frequencies of germaria containing one, two, and three GSCs, and average number of GSCs per germarium in virgin and mated females. For statistical analysis, a Mann-Whitney *U* test was used. *P* value is provided for comparison with control. *P* ≤ 0.05 was considered statistically significant (shown in bold). The number of germaria analyzed are shown in parentheses.
(PDF)

S2 Table Source data for the temporal change in number of germaria containing one, two or three GSCs in Fig 1H, Fig 1I and S1 Fig. Temporal change in frequencies of germaria containing one, two, and three GSCs, and average number of GSCs per germarium in virgin, mated and re-mated females. Females were mated with males 3 days after eclosion (1^st^ mating; +) and 7 days after the 1^st^ mating (2^nd^ mating; ++). For statistical analysis, a Mann-Whitney *U* test was used. *P* value is provided for comparison with control. *P* ≤ 0.05 was considered statistically significant (shown in bold). The number of germaria analyzed are shown in parentheses.
(PDF)

S1 Fig Temporal changes in the number of germline stem cells in virgin and mated females. (A, B) Frequencies of germaria containing one, two, and three GSCs (left y axis), and average number of GSCs per germarium (right y axis) in virgin (v), 1^st^ mated (m) and 2^nd^ mated (r) wild type female flies. The same data are represented in Fig 1H and 1I as line graphs. Three days after eclosion, females were mated with males. Mated females showed a significant increase in GSC number as compared with the virgin females until 6 days after the 1^st^ mating. Increase in GSC number occurred in the 2^nd^ mated females. For statistical analysis, a Mann-Whitney *U* test was used. ***P ≤ 0.001, **P ≤ 0.01, n.s., non-significant (*P* > 0.05).
(PDF)

S2 Fig The increase in GSC numbers is not associated with activation of egg laying of virgin females. (A) Number of laid eggs from virgin and mated female flies fed on yeast paste on grape juice-agar media in 48 hours. Box plot shows 25–75% (box), median (band inside) and minima to maxima (whiskers). (B) Frequencies of germaria containing one, two, and three GSCs (left y axis), and average number of GSCs per germarium (right y axis) in virgin and mated female flies. The female flies used were fed on yeast paste on grape juice-agar media (+ yeast) or standard cornmeal-agar-yeast media before mating. The numbers of germaria analyzed are shown inside bars. For statistical analysis, a Student’s t-test and a Mann-Whitney *U* test were used for A and B, respectively. **P ≤ 0.01, n.s., non-significant (*P* > 0.05).
(PDF)

S3 Fig Weight of ovaries in virgin and mated female flies. Each value is plotted as a point. Box plot shows 25–75% (box), median (band inside) and minima to maxima (whiskers). Ovaries from virgin females were heavier than those of mated females. For statistical analysis, a Student’s t-test was used. **P ≤ 0.01.
(PDF)

S4 Fig Expression pattern of *nvd* and sad in the ovariole. (A, B) Anti-Nvd and anti-Sad immunostaining in ovarioles in somatic follicle cell-specific RNAi for nvd and sad, respectively. (A) *yw* was crossed with *c587-GAL4* driver as a control. In *nvd* RNAi ovarioles, the anti-Nvd immunostaining signal was particularly reduced in stages 2–6 (left column) and stage 10 (right column) follicle cells. (B) Anti-sad immunostaining signal was reduced in stages 2–6 and stage 10 follicle cells of *sad* RNAi female flies. Inset of anti-Sad immunostaining image is a light-field image of the same specimen. Scale bar represents 50 μm.
(PDF)

S5 Fig Ecdysteroid signaling in the germline cells is not required for mating-induced increase in GSC numbers. Frequencies of germaria containing one, two, and three GSCs (left y axis), and average number of GSCs per germarium (right y axis) in virgin (v) and mated (m) female flies. Knocking down ecdysteroidogenic enzyme genes *nvd* or *sad* or ecdysteroid receptor gene *EcR* in the germ cells (using *nos-GAL4*) had no effect on mating-induced increase in GSCs. The numbers of germaria analyzed are shown inside bars. For statistical analysis, a Mann-Whitney *U* test was used. **P ≤ 0.01, *P ≤ 0.05.
(PDF)

S6 Fig Insulin signaling is not required for the mating-induced increase in GSC. numbers. (A–C) Frequencies of germaria containing one, two, and three GSCs (left y axis), and average number of GSCs per germarium (right y axis) in virgin (v) and mated (m) female flies overexpressing an *InR RNAi* transgene or a dominant-negative form of *InR* in the ovarian somatic cells. Transgenes were driven by *c587-GAL4*. The female flies used were aged for 4 days (A) and 10 days (B). A mating-induced increase in GSC numbers occurred even in loss of *InR* females that showed age-dependent GSC loss (B). (C) Female flies fed protein-free diet also showed a significant increase in GSC number after mating. Female flies were fed grape juice-agar media with yeast paste (control) or not (protein-free diet). The numbers of germaria analyzed are shown inside bars. For statistical analysis, a Mann-Whitney *U* test was used. ***P ≤ 0.001, **P ≤ 0.01, *P ≤ 0.05.
(PDF)

S7 Fig JH signaling is not required for the mating-induced increase in GSC numbers. (A–C) Frequencies of germaria containing one, two, and three GSCs (left y axis), and average number of GSCs per germarium (right y axis) in virgin (v) and mated (m) female flies. (A) Oral administration of methoprene, a JH analogue (JHa) to virgin and mated females. (B) Blocking endogenous JH production by knocking down of *juvenile hormone acid O-methyltransferase* (*JHAMT*) or overexpression of the protein phosphatase inhibitor *NiPp1* in the corpora allata (using *Aug21-Gal4)*. (C) Knocking down JH receptor *Met*, *gce* or JH target *Kr-h1* in the ovarian somatic cells (using *c587-GAL4*). The numbers of germaria analyzed are shown inside bars. For statistical analysis, a Mann-Whitney *U* test was used. ***P ≤ 0.001, **P ≤ 0.01, *P ≤ 0.05, n.s., non-significant (*P* > 0.05).
(PDF)

## References

[1] Wolfner MF. Battle and ballet: molecular interactions between the sexes in *Drosophila*. J Hered. 2009;100: 399–410. 10.1093/jhered/esp013 19349638 PMC2877532

[2] Spradling A, Drummond-Barbosa D, Kai T. Stem cells find their niche. Nature. 2001;414: 98–104. 11689954 10.1038/35102160

[3] Spradling A, Fuller MT, Braun RE, Yoshida S. Germline stem cells. Cold Spring Harb Perspect Biol. 2011;3: a002642 10.1101/cshperspect.a002642 21791699 PMC3220357

[4] Kai T, Spradling A. An empty *Drosophila* stem cell niche reactivates the proliferation of ectopic cells. Proc Natl Acad Sci U S A. 2003;100: 4633–4638. 12676994 10.1073/pnas.0830856100 PMC153607

[5] Manning A. A sperm factor affecting the receptivity of *Drosophila melanogaster* females. Nature. 1962;194: 252–253.

[6] Manning A. The control of sexual receptivity in female *Drosophila*. Anim Behav. 1967;15: 239–250. 6030948 10.1016/0003-3472(67)90006-1

[7] Kubli E. Sex-peptides: seminal peptides of the *Drosophila* male. Cell Mol Life Sci. 2003;60: 1689–1704. 14504657 10.1007/s00018-003-3052 PMC11146071

[8] Yapici N, Kim Y-J, Ribeiro C, Dickson BJ. A receptor that mediates the post-mating switch in *Drosophila* reproductive behaviour. Nature. 2008;451: 33–37. 18066048 10.1038/nature06483

[9] Häsemeyer M, Yapici N, Heberlein U, Dickson BJ. Sensory neurons in the *Drosophila* genital tract regulate female reproductive behavior. Neuron. 2009;61: 511–518. 10.1016/j.neuron.2009.01.009 19249272

[10] Rezával C, Pavlou HJ, Dornan AJ, Chan Y-B, Kravitz E a, Goodwin SF. Neural circuitry underlying *Drosophila* female postmating behavioral responses. Curr Biol. 2012;22: 1155–1165. 10.1016/j.cub.2012.04.062 22658598 PMC3396843

[11] Grueber WB, Ye B, Yang C-H, Younger S, Borden K, Jan LY, et al Projections of *Drosophila* multidendritic neurons in the central nervous system: links with peripheral dendrite morphology. Development. 2007;134: 55–64. 17164414 10.1242/dev.02666

[12] Hayashi S, Ito K, Sado Y, Taniguchi M, Akimoto A, Takeuchi H, et al GETDB, a database compiling expression patterns and molecular locations of a collection of Gal4 enhancer traps. Genesis. 34: 58–61. 12324948 10.1002/gene.10137

[13] Kimura K-I, Hachiya T, Koganezawa M, Tazawa T, Yamamoto D. Fruitless and Doublesex coordinate to generate male-specific neurons that can initiate courtship. Neuron. 2008;59: 759–769. 10.1016/j.neuron.2008.06.007 18786359

[14] Luo L, Liao YJ, Jan LY, Jan YN. Distinct morphogenetic functions of similar small GTPases: *Drosophila* Drac1 is involved in axonal outgrowth and myoblast fusion. Genes Dev. 1994;8: 1787–1802. 7958857 10.1101/gad.8.15.1787

[15] Yang C-H, Rumpf S, Xiang Y, Gordon MD, Song W, Jan LY, et al Control of the postmating behavioral switch in *Drosophila* females by internal sensory neurons. Neuron. 2009;61: 519–526. 10.1016/j.neuron.2008.12.021 19249273 PMC2748846

[16] Nakayama S, Kaiser K, Aigaki T. Ectopic expression of sex-peptide in a variety of tissues in *Drosophila* females using the P[GAL4] enhancer-trap system. Mol Gen Genet. 1997;254: 449–455. 9180699 10.1007/s004380050438

[17] Gou B, Liu Y, Guntur AR, Stern U, Yang C-H. Mechanosensitive neurons on the internal reproductive tract contribute to egg-laying-induced acetic Acid attraction in *Drosophila*. Cell Rep. 2014;9: 522–530. 10.1016/j.celrep.2014.09.033 25373900 PMC4223655

[18] Ables ET, Drummond-Barbosa D. The steroid hormone ecdysone functions with intrinsic chromatin remodeling factors to control female germline stem cells in *Drosophila*. Cell Stem Cell. 2010;7: 581–592. 10.1016/j.stem.2010.10.001 21040900 PMC3292427

[19] König A, Yatsenko AS, Weiss M, Shcherbata HR. Ecdysteroids affect *Drosophila* ovarian stem cell niche formation and early germline differentiation. EMBO J. 2011;30: 1549–1562. 10.1038/emboj.2011.73 21423150 PMC3102283

[20] Morris LX, Spradling AC. Steroid signaling within *Drosophila* ovarian epithelial cells sex-specifically modulates early germ cell development and meiotic entry. PLOS One. 2012;7: e46109 10.1371/journal.pone.0046109 23056242 PMC3462805

[21] Ables ET, Bois KE, Garcia CA, Drummond-Barbosa D. Ecdysone response gene *E78* controls ovarian germline stem cell niche formation and follicle survival in *Drosophila*. Dev Biol. 2015;400: 33–42. 10.1016/j.ydbio.2015.01.013 25624267 PMC4448935

[22] Uryu O, Ameku T, Niwa R. Recent progress in understanding the role of ecdysteroids in adult insects: Germline development and circadian clock in the fruit fly *Drosophila melanogaster*. Zool Lett. 2015;1: 32 10.1186/s40851-015-0031-2 PMC4657291 26605077

[23] Harshman LG, Loeb AM, Johnson BA. Ecdysteroid titers in mated and unmated *Drosophila melanogaster* females. J Insect Physiol. 1999;45: 571–577. 10.1016/S0022-1910(99)00038-4 12770342

[24] Yoshiyama T, Namiki T, Mita K, Kataoka H, Niwa R. Neverland is an evolutionally conserved Rieske-domain protein that is essential for ecdysone synthesis and insect growth. Development. 2006;133: 2565–2574. 10.1242/dev.02428 16763204

[25] Yoshiyama-Yanagawa T, Enya S, Shimada-Niwa Y, Yaguchi S, Haramoto Y, Matsuya T, et al The conserved Rieske oxygenase DAF-36/Neverland is a novel cholesterol-metabolizing enzyme. J Biol Chem. 2011;286: 25756–25762. 10.1074/jbc.M111.244384 21632547 PMC3138242

[26] Warren JT, Petryk A, Marque G, Jarcho M, Parvy J-P, Dauphin-villemant C, et al Molecular and biochemical characterization of two P450 enzymes in the ecdysteroidogenic pathway of *Drosophila melanogaster*. Proc Natl Acad Sci U S A. 2002;99: 11043–11048. 12177427 10.1073/pnas.162375799 PMC123207

[27] Drummond-Barbosa D, Spradling AC. Stem cells and their progeny respond to nutritional changes during *Drosophila* oogenesis. Dev Biol. 2001;231: 265–278. 11180967 10.1006/dbio.2000.0135

[28] LaFever L, Drummond-Barbosa D. Direct control of germline stem cell division and cyst growth by neural insulin in *Drosophila*. Science. 2005;309: 1071–1073. 16099985 10.1126/science.1111410

[29] Hsu H-J, Drummond-Barbosa D. Insulin levels control female germline stem cell maintenance via the niche in *Drosophila*. Proc Natl Acad Sci U S A. 2009;106: 1117–1121. 10.1073/pnas.0809144106 19136634 PMC2633547

[30] Soller M, Bownes M, Kubli E. Control of oocyte maturation in sexually mature *Drosophila* females. Dev Biol. 1999;208: 337–351. 10191049 10.1006/dbio.1999.9210

[31] Parker L, Gross S, Beullens M, Bollen M, Bennett D, Alphey L. Functional interaction between nuclear inhibitor of protein phosphatase type 1 (NIPP1) and protein phosphatase type 1 (PP1) in *Drosophila*: consequences of over-expression of NIPP1 in flies and suppression by co-expression of PP1. Biochem J. 2002;368: 789–797. 12358598 10.1042/BJ20020582 PMC1223049

[32] Reiff T, Jacobson J, Cognigni P, Antonello Z, Ballesta E, Tan KJ, et al Endocrine remodelling of the adult intestine sustains reproduction in *Drosophila*. Elife. 2015;4: e06930 10.7554/eLife.06930 26216039 PMC4515472

[33] Niwa R, Niimi T, Honda N, Yoshiyama M, Itoyama K, Kataoka H, et al Juvenile hormone acid *O*-methyltransferase in *Drosophila melanogaster*. Insect Biochem Mol Biol. 2008;38: 714–720. 10.1016/j.ibmb.2008.04.003 18549957

[34] Jindra M, Palli SR, Riddiford LM. The juvenile hormone signaling pathway in insect development. Annu Rev Entomol. 2013;58: 181–204. 10.1146/annurev-ento-120811-153700 22994547

[35] Niwa YS, Niwa R. Transcriptional regulation of insect steroid hormone biosynthesis and its role in controlling timing of molting and metamorphosis. Development Growth and Differentiation. 2015. 10.1111/dgd.12248 PMC11520982 26667894

[36] Feng K, Palfreyman MT, Häsemeyer M, Talsma A, Dickson BJ. Ascending SAG neurons control sexual receptivity of *Drosophila* females. Neuron. 2014;83: 135–148. 10.1016/j.neuron.2014.05.017 24991958

[37] Hussain A, Üçpunar HK, Zhang M, Loschek LF, Grunwald Kadow IC. Neuropeptides modulate female chemosensory processing upon mating in *Drosophila*. PLOS Biol. 2016;14: e1002455 10.1371/journal.pbio.1002455 27145127 PMC4856363

[38] Haussmann IU, Hemani Y, Wijesekera T, Dauwalder B, Soller M. Multiple pathways mediate the sex-peptide-regulated switch in female *Drosophila* reproductive behaviours. Proc Biol Sci. 2013;280: 20131938 10.1098/rspb.2013.1938 24089336 PMC3790487

[39] Moshitzky P, Fleischmann I, Chaimov N, Saudan P, Klauser S, Kubli E, et al Sex-peptide activates juvenile hormone biosynthesis in the *Drosophila melanogaster* corpus allatum. Arch Insect Biochem Physiol. 1996;32: 363–374. 10.1002/(SICI)1520-6327(1996)32:3/4<363::AID-ARCH9>3.0.CO;2-T 8756302

[40] Bontonou G, Shaik HA, Denis B, Wicker-Thomas C. Acp70A regulates *Drosophila* pheromones through juvenile hormone induction. Insect Biochem Mol Biol. 2015;56: 36–49. 10.1016/j.ibmb.2014.11.008 25484200

[41] Niwa YS, Niwa R. Neural control of steroid hormone biosynthesis during development in the fruit fly *Drosophila melanogaster*. Genes Genet Syst. 2014;89: 27–34. 24817759 10.1266/ggs.89.27

[42] Shimada-Niwa Y, Niwa R. Serotonergic neurons respond to nutrients and regulate the timing of steroid hormone biosynthesis in *Drosophila*. Nat Commun. 2014;5: 5778 10.1038/ncomms6778 25502946 PMC4284655

[43] Ohhara Y, Shimada-Niwa Y, Niwa R, Kayashima, Hayashi Y, Akagi K, et al Autocrine regulation of ecdysone synthesis by β3-octopamine receptor in the prothoracic gland is essential for *Drosophila* metamorphosis. Proc Natl Acad Sci U S A. 2015;112: 1452–1457. 10.1073/pnas.1414966112 25605909 PMC4321272

[44] Yamanaka N, Hua Y-J, Roller L, Spalovska-Valachova I, Mizoguchi A, Kataoka H, et al *Bombyx* prothoracicostatic peptides activate the sex peptide receptor to regulate ecdysteroid biosynthesis. Proc Natl Acad Sci. 2010;107: 2060–2065. 10.1073/pnas.0907471107 20133850 PMC2836647

[45] Riehle MA, Brown MR. Insulin stimulates ecdysteroid production through a conserved signaling cascade in the mosquito *Aedes aegypti*. Insect Biochem Mol Biol. 1999;29: 855–860. 10528406 10.1016/s0965-1748(99)00084-3

[46] Brown MR, Clark KD, Gulia M, Zhao Z, Garczynski SF, Crim JW, et al An insulin-like peptide regulates egg maturation and metabolism in the mosquito *Aedes aegypti*. Proc Natl Acad Sci U S A. 2008;105: 5716–5721. 10.1073/pnas.0800478105 18391205 PMC2311378

[47] Brown MR, Graf R, Swiderek KM, Fendley D, Stracker TH, Champagne DE, et al Identification of a steroidogenic neurohormone in female mosquitoes. J Biol Chem. 1998;273: 3967–3971. 9461584 10.1074/jbc.273.7.3967

[48] Vogel KJ, Brown MR, Strand MR. Ovary ecdysteroidogenic hormone requires a receptor tyrosine kinase to activate egg formation in the mosquito *Aedes aegypti*. Proc Natl Acad Sci U S A. 2015;112: 5057–5062. 10.1073/pnas.1501814112 25848040 PMC4413300

[49] Veenstra JA. What the loss of the hormone neuroparsin in the melanogaster subgroup of *Drosophila* can tell us about its function. Insect Biochem Mol Biol. 2010;40: 354–361. 10.1016/j.ibmb.2010.03.001 20226240

[50] Nakada D, Levi BP, Morrison SJ. Integrating physiological regulation with stem cell and tissue homeostasis. Neuron. 2011;70: 703–718. 10.1016/j.neuron.2011.05.011 21609826 PMC4521627

[51] Petrulis A. Chemosignals, hormones and mammalian reproduction. Horm Behav. 2013;63: 723–741. 10.1016/j.yhbeh.2013.03.011 23545474 PMC3667964

[52] Santel A, Winhauer T, Blümer N, Renkawitz-Pohl R. The *Drosophila don juan (dj)* gene encodes a novel sperm specific protein component characterized by an unusual domain of a repetitive amino acid motif. Mech Dev. 1997;64: 19–30. 9232593 10.1016/s0925-4773(97)00031-2

[53] Bender M, Imam FB, Talbot WS, Ganetzky B, Hogness DS. *Drosophila* ecdysone receptor mutations reveal functional differences among receptor isoforms. Cell. 1997;91: 777–788. 9413987 10.1016/s0092-8674(00)80466-3

[54] Liu H, Kubli E. Sex-peptide is the molecular basis of the sperm effect in *Drosophila melanogaster*. Proc Natl Acad Sci U S A. 2003;100: 9929–9933. 12897240 10.1073/pnas.1631700100 PMC187889

[55] Zhu C-H, Xie T. Clonal expansion of ovarian germline stem cells during niche formation in *Drosophila*. Development. 2003;130: 2579–2588. 12736203 10.1242/dev.00499

[56] Kai T, Spradling A. Differentiating germ cells can revert into functional stem cells in *Drosophila melanogaster* ovaries. Nature. 2004;428: 564–569. 15024390 10.1038/nature02436

[57] Siegmund T, Korge G. Innervation of the ring gland of *Drosophila melanogaster*. J Comp Neurol. 2001;431: 481–491. d 11223816 10.1002/1096-9861(20010319)431:4<481::aid-cne1084>3.0.co;2-7

[58] Ding D, Parkhurst SM, Lipshitz HD. Different genetic requirements for anterior RNA localization revealed by the distribution of Adducin-like transcripts during *Drosophila* oogenesis. Proc Natl Acad Sci U S A. 1993;90: 2512–2516. 7681599 10.1073/pnas.90.6.2512 PMC46118

[59] Oda H, Uemura T, Harada Y, Iwai Y, Takeichi M. A *Drosophila* homolog of cadherin associated with armadillo and essential for embryonic cell-cell adhesion. Dev Biol. 1994;165: 716–726. 7958432 10.1006/dbio.1994.1287

[60] Riemer D, Stuurman N, Berrios M, Hunter C, Fisher PA, Weber K. Expression of *Drosophila* lamin C is developmentally regulated: analogies with vertebrate A-type lamins. J Cell Sci. 1995;108: 3189–3198. 7593280 10.1242/jcs.108.10.3189

[61] Gibbens YY, Warren JT, Gilbert LI, O’Connor MB. Neuroendocrine regulation of *Drosophila* metamorphosis requires TGFβ/Activin signaling. Development. 2011;138: 2693–2703. 10.1242/dev.063412 21613324 PMC3109597

[62] Pankotai T, Popescu C, Martín D, Grau B, Zsindely N, Bodai L, et al Genes of the ecdysone biosynthesis pathway are regulated by the dATAC histone acetyltransferase complex in *Drosophila*. Mol Cell Biol. 2010;30: 4254–4266. 10.1128/MCB.00142-10 20584983 PMC2937542

[63] Yamanaka N, Marqués G, O’Connor MB. Vesicle-mediated steroid hormone secretion in *Drosophila melanogaster*. Cell. 2015;163: 907–919. 10.1016/j.cell.2015.10.022 26544939 PMC4636736

[64] McBrayer Z, Ono H, Shimell M, Parvy J-P, Beckstead RB, Warren JT, et al Prothoracicotropic hormone regulates developmental timing and body size in *Drosophila*. Dev Cell. 2007;13: 857–871. 18061567 10.1016/j.devcel.2007.11.003 PMC2359579

[65] Niwa R, Namiki T, Ito K, Shimada-Niwa Y, Kiuchi M, Kawaoka S, et al *Non-molting glossy/shroud* encodes a short-chain dehydrogenase/reductase that functions in the “Black Box” of the ecdysteroid biosynthesis pathway. Development. 2010;137: 1991–1999. 10.1242/dev.045641 20501590

[66] Enya S, Ameku T, Igarashi F, Iga M, Kataoka H, Shinoda T, et al A Halloween gene *noppera-bo* encodes a glutathione *S*-transferase essential for ecdysteroid biosynthesis via regulating the behaviour of cholesterol in *Drosophila*. Sci Rep. 2014;4: 6586 10.1038/srep06586 25300303 PMC4192634

