# Supplementary material for: Mating-Induced Increase in Germline Stem Cells via the Neuroendocrine System in Female Drosophila
Source: PLoS Genet. 2016 Jun 16;12(6):e1006123. doi: 10.1371/journal.pgen.1006123 (PMC4911108; doi:10.1371/journal.pgen.1006123)
Supplement: S1 Table — Frequencies of germaria containing one, two, and three GSCs, and average number of GSCs per germarium in virgin and mated females. For statistical analysis, a Mann-Whitney U test was used. P value is provided for comparison with control. P ≤ 0.05 was considered statistically significant (shown in bold). The number of germaria analyzed are shown in parentheses. (PDF) [file pgen.1006123.s001.pdf]

S1 Table

Genotype	Mating	Number of germaria			Average # of GSCs	*p* Value	Control	Figure panel
		3 GSCs	2 GSCs	1 GSCs				
yw wild-type	-	22	52	10	2.14 (84)	**0.0009**		Fig 1B
	+	46	48	3	2.44 (97)			
yw wild-type	-	15	28	7	2.16 (50)			Fig 2A
	+	25	25	0	2.50 (50)	**0.0087**	*yw* wild-type (-)	
	+ $SP^{0/\Delta}$	12	31	7	2.10 (50)	0.6129		
yw wild-type	-	11	35	4	2.14 (84)	**0.0042**		Fig 2B
	+	24	25	1	2.46 (97)			
$SPR^{Df(1)}$	-	11	35	4	2.14 (84)	**0.0042**		
	+	24	25	1	2.46 (97)			
elav / +	-	11	37	2	2.18 (50)	**0.007**		Fig 2C
	+	23	27	0	2.46 (50)			
elav > SPR RNAi	-	12	37	1	2.22 (50)	0.5		
	+	16	32	2	2.28 (50)			
ppk / +	-	29	72	7	2.20 (108)	**0.0025**		
	+	45	55	0	2.45 (100)			
ppk > mSP	-	24	28	0	2.46 (52)	**0.0021**	*ppk / +* (-)	
ppk > SPR RNAi	-	12	35	3	2.18 (50)	0.87		
	+	12	36	2	2.20 (50)			
NP21 / +	-	30	72	6	2.22 (108)	**0.002**		
	+	46	54	0	2.46 (100)			
NP21 > mSP	-	28	28	0	2.50 (56)	**0.0021**	*NP21 / +* (-)	
NP21 > SPR RNAi	-	13	35	2	2.22 (50)	0.66		
	+	16	31	3	2.26 (50)			
c587 / +	-	12	35	3	2.18 (50)	**0.0168**		Fig 2E
	+	23	26	1	2.44 (50)			
c587 > mSP	-	12	30	8	2.08 (50)	0.7372	*c587 / +* (-)	
c587 > SPR RNAi	-	10	37	3	2.14 (50)	**0.0354**		
	+	20	28	2	2.36 (50)			
yw (standard food)	-	12	34	4	2.16 (50)			S2B Fig
yw (yeast food)	+	24	26	0	2.48 (50)	**0.0015**	*yw* (standard food) (-)	
	-	14	33	3	2.22 (50)	0.5428		

(continued on next page)

Genotype	Mating	Number of germaria			Average # of GSCs	*p* Value	Control	Figure panel
		3 GSCs	2 GSCs	1 GSCs				
c587 / +	-	19	51	5	2.19 (75)	**0.0082**		Fig 3B
	+	32	43	0	2.43 (75)			
c587 > nvd RNAi	-	19	51	6	2.16 (75)	0.38		
	+	21	51	3	2.24 (75)			
c587 > nvd RNAi; nvd-Bm[wt]	-	15	51	9	2.08 (75)	**0.0007**		
	+	31	43	1	2.40 (75)			
c587 > nvd RNAi; nvd-Bm[H190A]	-	17	48	10	2.09 (75)	0.34		
	+	20	49	6	2.19 (75)			
EcR^{A483T} or EcR^{M554fs} / +	-	9	32	9	2.00 (50)	**0.0067**		Fig 3E
	+	21	25	4	2.34 (50)			
EcR^{A483T} / EcR^{M554fs}	-	10	28	12	1.96 (50)	0.45		
	+	5	33	12	1.86 (50)			
c587 / +	-	15	29	6	2.18 (50)	**0.014**		
	+	25	24	1	2.48 (50)			
c587 > EcR RNAi	-	11	36	3	2.16 (50)	0.9		
	+	12	33	5	2.14 (50)			
c587 / + (EtOH)	-	11	35	4	2.14 (50)	**0.0042**		Fig 3F
	+	24	25	1	2.46 (50)			
c587 > nvd RNAi (EtOH)	-	10	36	4	2.12 (50)	0.43		
	+	15	30	5	2.20 (50)			
c587 > nvd RNAi (7dC)	-	21	69	10	2.11 (100)	**0.04**		
	+	34	66	3	2.37 (103)			
c587 > nvd RNAi (20E)	-	12	55	8	2.05 (75)	**0.0000**		
	+	33	42	0	2.44 (75)			
c587 > sad RNAi (EtOH)	-	16	49	10	2.08 (75)	0.59		
	+	15	55	5	2.13 (75)			
c587 > sad RNAi (20E)	-	17	53	5	2.16 (75)	**0.0027**		
	+	33	41	1	2.43 (75)			
nos / +	-	14	29	7	2.14 (50)	**0.01226**		S5 Fig
	+	23	27	0	2.46 (50)			
nos > nvd RNAi	-	12	31	7	2.10 (50)	**0.005942**		
	+	25	25	0	2.50 (50)			
nos > sad RNAi	-	16	26	8	2.16 (50)	**0.01799**		
	+	25	24	1	2.48 (50)			
nos > EcR RNAi	-	12	31	7	2.10 (50)	**0.0135**		
	+	25	25	0	2.50 (50)			
c587 / + (4-day-old)	-	16	43	6	2.15 (65)	**0.0039**		S6A Fig
	+	29	36	0	2.45 (65)			
c587 > InR RNAi (4-day-old)	-	11	43	11	2.00 (65)	**0.0067**		
	+	20	43	2	2.28 (65)			
c587 > InR DN (4-day-old)	-	15	40	10	2.08 (65)	**0.0305**		
	+	22	40	3	2.29 (65)			

(continued on next page)

Genotype	Mating	Number of germaria			Average # of GSCs	*p* Value	Control	Figure panel
		3 GSCs	2 GSCs	1 GSCs				
c587 / + (10-day-old)	-	18	42	5	2.20 (65)			S6B Fig
c587 > InR RNAi (10-day-old)	-	5	39	16	1.82 (60)	**0.0003**	*c587 / +* (10-day-old)	
c587 > InR DN (10-day-old)	-	7	47	6	2.02 (60)	**0.0457**	*c587 / +* (10-day-old)	
yw (+control diet)	-	13	40	2	2.20 (50)	**0.01663**		S6C Fig
	+	24	31	0	2.44 (50)			
yw (+protein-free diet)	-	13	39	3	2.18 (50)	**0.007889**		
	+	25	30	0	2.45 (50)			
yw wild-type (EtOH)	-	16	26	8	2.16 (50)	0.01799		S7A Fig
	+	25	24	1	2.48 (50)			
yw wild-type (JHa)	-	15	32	3	2.24 (50)	0.02218		
	+	25	25	0	2.50 (50)			
Aug21 / +	-	11	36	3	2.16 (50)	**0.0091**		S7B Fig
	+	22	28	0	2.44 (50)			
Aug21 > JHAMT RNAi	-	12	35	3	2.18 (50)	**0.0424**		
	+	20	30	0	2.40 (50)			
Aug21 > NiPp1	-	12	40	8	2.07 (60)	**0.003379**		
	+	25	33	2	2.38 (60)			
c587 / +	-	12	32	6	2.12 (50)	**0.004643**		S7C Fig
	+	23	27	0	2.46 (50)			
c587 > Met RNAi	-	9	32	9	2.00 (50)	**0.000587**		
	+	21	29	0	2.42 (50)			
c587 > Gce RNAi	-	10	31	9	2.02 (50)	**0.0026**		
	+	22	26	2	2.40 (50)			
c587 > Met RNAi	-	10	33	7	2.06 (50)	**0.000825**		
	+	23	27	0	2.46 (50)			
ppk / + (EtOH)	-	17	31	2	2.30 (50)	**0.0031**		Fig 5C
	+	24	26	0	2.48 (50)			
ppk > SPR RNAi (EtOH)	-	15	34	1	2.28 (50)	0.83		
	+	14	35	1	2.26 (50)			
ppk > SPR RNAi (20E)	-	13	34	3	2.20 (50)	**0.021**		
	+	24	26	0	2.48 (50)			
ppk / + (20E)	-	15	34	1	2.28 (50)	**0.013**		
	+	27	23	0	2.48 (50)			
NP21 / + (EtOH)	-	15	33	2	2.54 (50)	**0.017**		
	+	26	24	0	2.26 (50)			
NP21 > SPR RNAi (EtOH)	-	14	33	3	2.52 (50)	0.92		
	+	15	30	5	2.22 (50)			
NP21 > SPR RNAi (20E)	-	14	32	4	2.20 (50)	**0.04**		
	+	22	28	0	2.44 (50)			
NP21 / + (20E)	-	15	33	1	2.24 (50)	**0.021**		
	+	24	26	1	2.50 (50)			

(continued on next page)

Genotype	Mating	Number of germaria			Average # of GSCs	*p* Value	Control	Figure panel
		3 GSCs	2 GSCs	1 GSCs				
yw wild-type (EtOH)	-	18	35	7	2.18 (60)	**0.005624**		Fig 5D
	+	30	30	0	2.50 (60)			
yw wild-type (EtOH)	-	19	39	2	2.28 (60)	0.421	*yw* wild-type (EtOH)	
yw wild-type (20E)	+	17	40	3	2.23 (60)	0.724	*yw* wild-type (EtOH)	
yw wild-type (20E)	+ $SP^{0/\Delta}$	19	35	6	2.22 (60)	0.7765	*yw* wild-type (EtOH)	
