# Supplementary material for: Mating-Induced Increase in Germline Stem Cells via the Neuroendocrine System in Female Drosophila
Source: PLoS Genet. 2016 Jun 16;12(6):e1006123. doi: 10.1371/journal.pgen.1006123 (PMC4911108; doi:10.1371/journal.pgen.1006123)
Supplement: S2 Table — Temporal change in frequencies of germaria containing one, two, and three GSCs, and average number of GSCs per germarium in virgin, mated and re-mated females. Females were mated with males 3 days after eclosion (1st mating; +) and 7 days after the 1st mating (2nd mating; ++). For statistical analysis, a Mann-Whitney U test was used. P value is provided for comparison with control. P ≤ 0.05 was considered statistically significant (shown in bold). The number of germaria analyzed are shown in parentheses. (PDF) [file pgen.1006123.s002.pdf]

S2 Table

For Fig 1H

Genotype	Mating	Day after mating	Day after eclosion	Number of germaria			Average # of GSCs	*p* Value
				3 GSCs	2 GSCs	1 GSCs		
yw wild-type	-		1	18	31	1	2.34 (50)	
	-		2	17	31	2	2.30 (50)	
	-		3	14	33	3	2.22 (50)	
	-		4	16	54	5	2.15 (75)	**0.0014**
	+	1		32	43	0	2.43 (75)	
	-		5	17	51	7	2.13 (75)	**0.0041**
	+	2		32	41	2	2.40 (75)	
	-		6	14	52	9	2.07 (75)	**0.0018**
	+	3		31	40	4	2.36 (75)	
	-		7	13	52	10	2.04 (75)	**0.0008**
	+	4		31	40	4	2.36 (75)	
	-		8	13	49	13	2.00 (75)	**0.0013**
	+	5		24	50	1	2.31 (75)	
	-		9	12	47	16	1.95 (75)	**0.0001**
	+	6		28	44	3	2.33 (75)	
	-		10	10	51	14	1.95 (75)	0.0843
	+	7		16	51	8	2.11 (75)	
	-		11	13	52	10	2.04 (75)	control
	+	8		15	48	12	2.04 (75)	0.9892
	++	1		33	40	2	2.41 (75)	**0.0001**

(continued on next page)

For Fig 1I

Genotype	Mating	Day after mating	Day after eclosion	Number of germaria			Average # of GSCs	*p* Value
				3 GSCs	2 GSCs	1 GSCs		
yw wild-type	-		3	19	26	5	2.28 (50)	
	-		4	13	34	3	2.20 (50)	**0.04646**
	+	1		21	29	0	2.42 (50)	
	-		10	8	34	8	2.00 (50)	0.6074
	+	7		11	31	8	2.06 (50)	
	-		11	7	31	12	1.90 (50)	control
	+	8		9	31	10	1.98 (50)	0.5182
	++	1		25	24	1	2.48 (50)	**8.108e-06**
	-		12	7	30	13	1.88 (50)	control
	+	9		7	32	11	1.92 (50)	0.7313
	++	2		24	25	1	2.46 (50)	**1.037e-05**
	-		13	8	27	15	1.86 (50)	control
	+	10		8	30	12	1.92 (50)	0.6253
	++	3		20	27	3	2.34 (50)	**0.0004182**
	-		14	8	29	13	1.90 (50)	control
	+	11		5	37	8	1.94 (50)	0.6836
	++	4		20	27	3	2.34 (50)	**0.0008593**
	-		15	5	34	11	1.88 (50)	control
	+	12		5	34	11	1.88 (50)	1
	++	5		22	21	7	2.30 (50)	**0.001228**
	-		16	7	27	16	1.82 (50)	control
	+	13		5	34	11	1.88 (50)	0.5589
	++	6		19	22	9	2.20 (50)	**0.008003**
	-		17	6	27	17	1.78 (50)	control
	+	14		4	33	13	1.82 (50)	0.6628
	++	7		9	30	11	1.96 (50)	0.1591
