# Supplementary figures and images for: Mating-Induced Increase in Germline Stem Cells via the Neuroendocrine System in Female Drosophila
Source: PLoS Genet. 2016 Jun 16;12(6):e1006123. doi: 10.1371/journal.pgen.1006123 (PMC4911108; doi:10.1371/journal.pgen.1006123)

S1 Fig

**A**

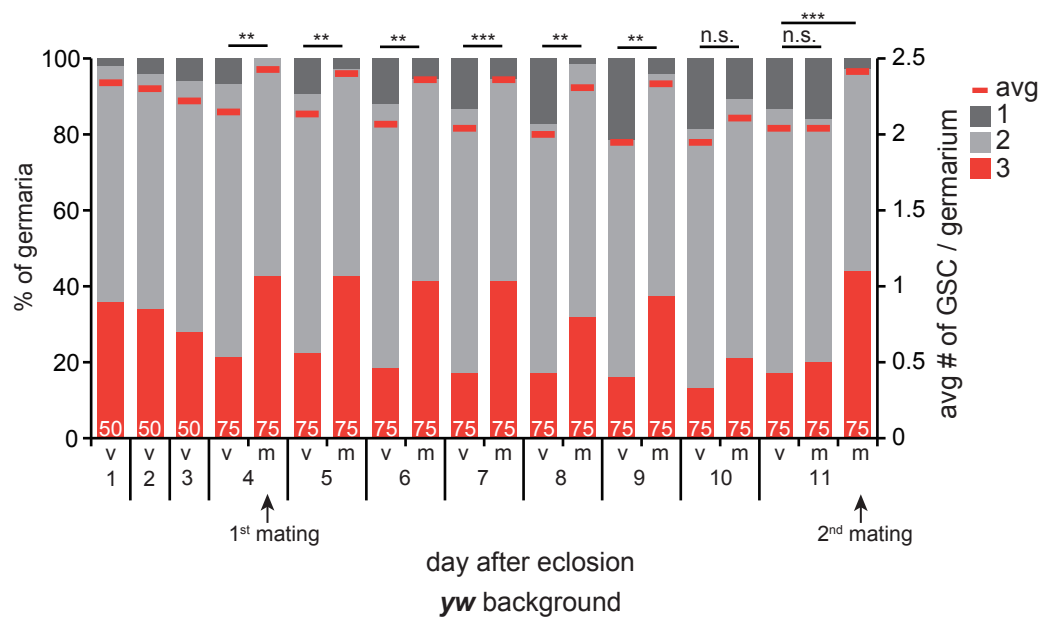

**B**

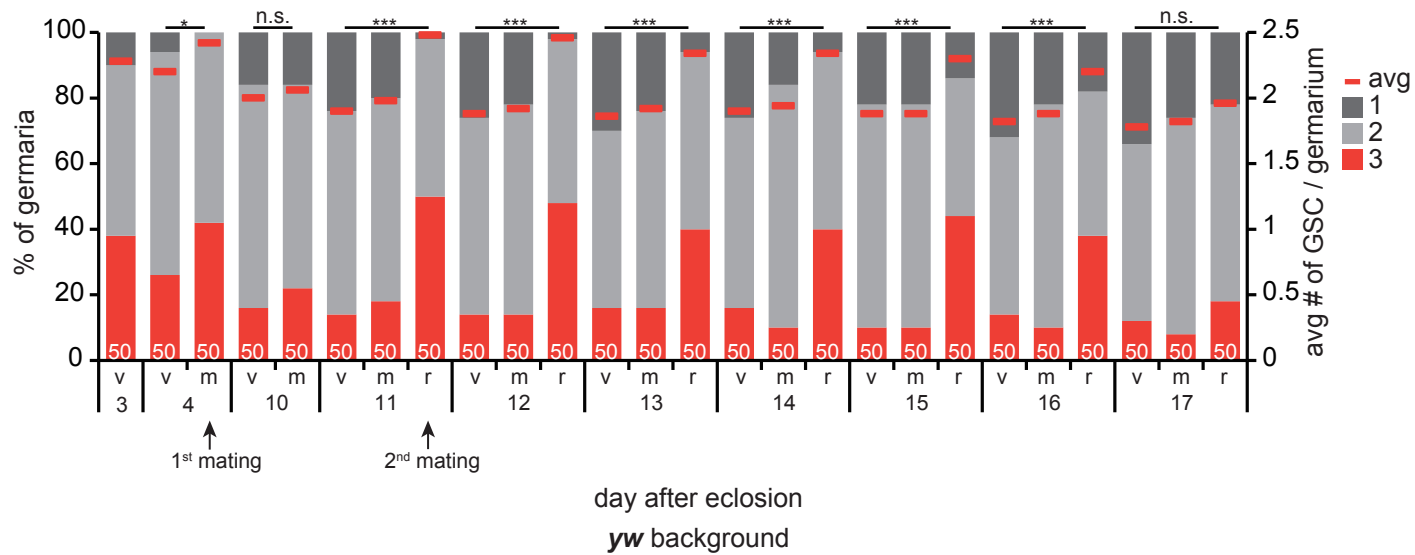

Supplement: S1 Fig — (A, B) Frequencies of germaria containing one, two, and three GSCs (left y axis), and average number of GSCs per germarium (right y axis) in virgin (v), 1st mated (m) and 2nd mated (r) wild type female flies. The same data are represented in Fig 1H and 1I as line graphs. Three days after eclosion, females were mated with males. Mated females showed a significant increase in GSC number as compared with the virgin females until 6 days after the 1st mating. Increase in GSC number occurred in the 2nd mated females. For statistical analysis, a Mann-Whitney U test was used. ***P ≤ 0.001, **P ≤ 0.01, n.s., non-significant (P > 0.05). (PDF) [file pgen.1006123.s003.pdf]

S2 Fig

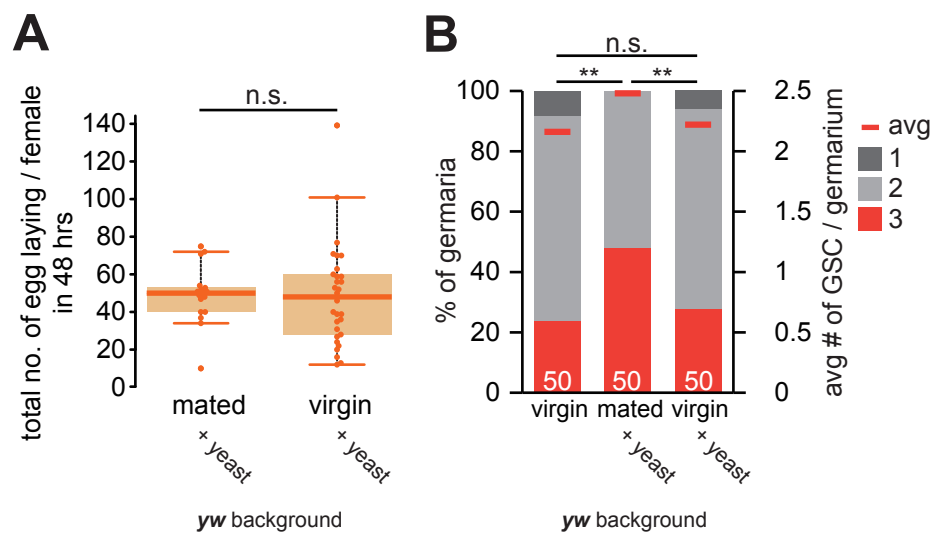

Supplement: S2 Fig — (A) Number of laid eggs from virgin and mated female flies fed on yeast paste on grape juice-agar media in 48 hours. Box plot shows 25–75% (box), median (band inside) and minima to maxima (whiskers). (B) Frequencies of germaria containing one, two, and three GSCs (left y axis), and average number of GSCs per germarium (right y axis) in virgin and mated female flies. The female flies used were fed on yeast paste on grape juice-agar media (+ yeast) or standard cornmeal-agar-yeast media before mating. The numbers of germaria analyzed are shown inside bars. For statistical analysis, a Student’s t-test and a Mann-Whitney U test were used for A and B, respectively. **P ≤ 0.01, n.s., non-significant (P > 0.05). (PDF) [file pgen.1006123.s004.pdf]

S3 Fig

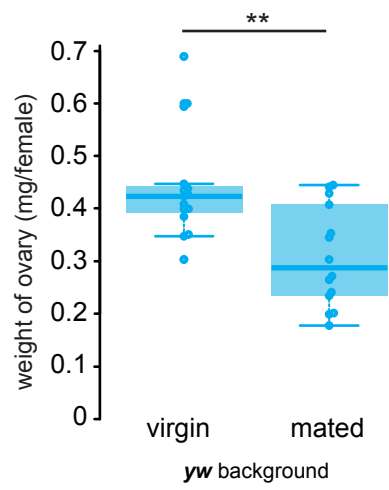

Supplement: S3 Fig — Each value is plotted as a point. Box plot shows 25–75% (box), median (band inside) and minima to maxima (whiskers). Ovaries from virgin females were heavier than those of mated females. For statistical analysis, a Student’s t-test was used. **P ≤ 0.01. (PDF) [file pgen.1006123.s005.pdf]

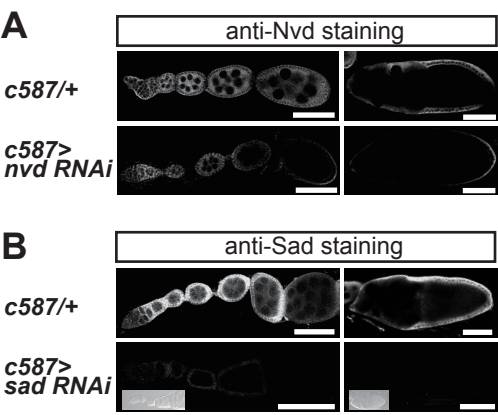

Supplement: S4 Fig — (A, B) Anti-Nvd and anti-Sad immunostaining in ovarioles in somatic follicle cell-specific RNAi for nvd and sad, respectively. (A) yw was crossed with c587-GAL4 driver as a control. In nvd RNAi ovarioles, the anti-Nvd immunostaining signal was particularly reduced in stages 2–6 (left column) and stage 10 (right column) follicle cells. (B) Anti-sad immunostaining signal was reduced in stages 2–6 and stage 10 follicle cells of sad RNAi female flies. Inset of anti-Sad immunostaining image is a light-field image of the same specimen. Scale bar represents 50 μm. (PDF) [file pgen.1006123.s006.pdf]

S5 Fig

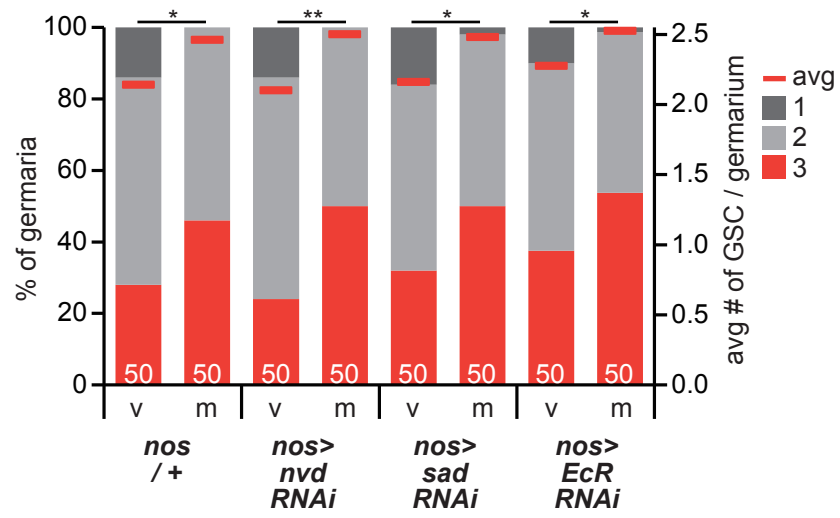

Supplement: S5 Fig — Frequencies of germaria containing one, two, and three GSCs (left y axis), and average number of GSCs per germarium (right y axis) in virgin (v) and mated (m) female flies. Knocking down ecdysteroidogenic enzyme genes nvd or sad or ecdysteroid receptor gene EcR in the germ cells (using nos-GAL4) had no effect on mating-induced increase in GSCs. The numbers of germaria analyzed are shown inside bars. For statistical analysis, a Mann-Whitney U test was used. **P ≤ 0.01, *P ≤ 0.05. (PDF) [file pgen.1006123.s007.pdf]

S6 Fig

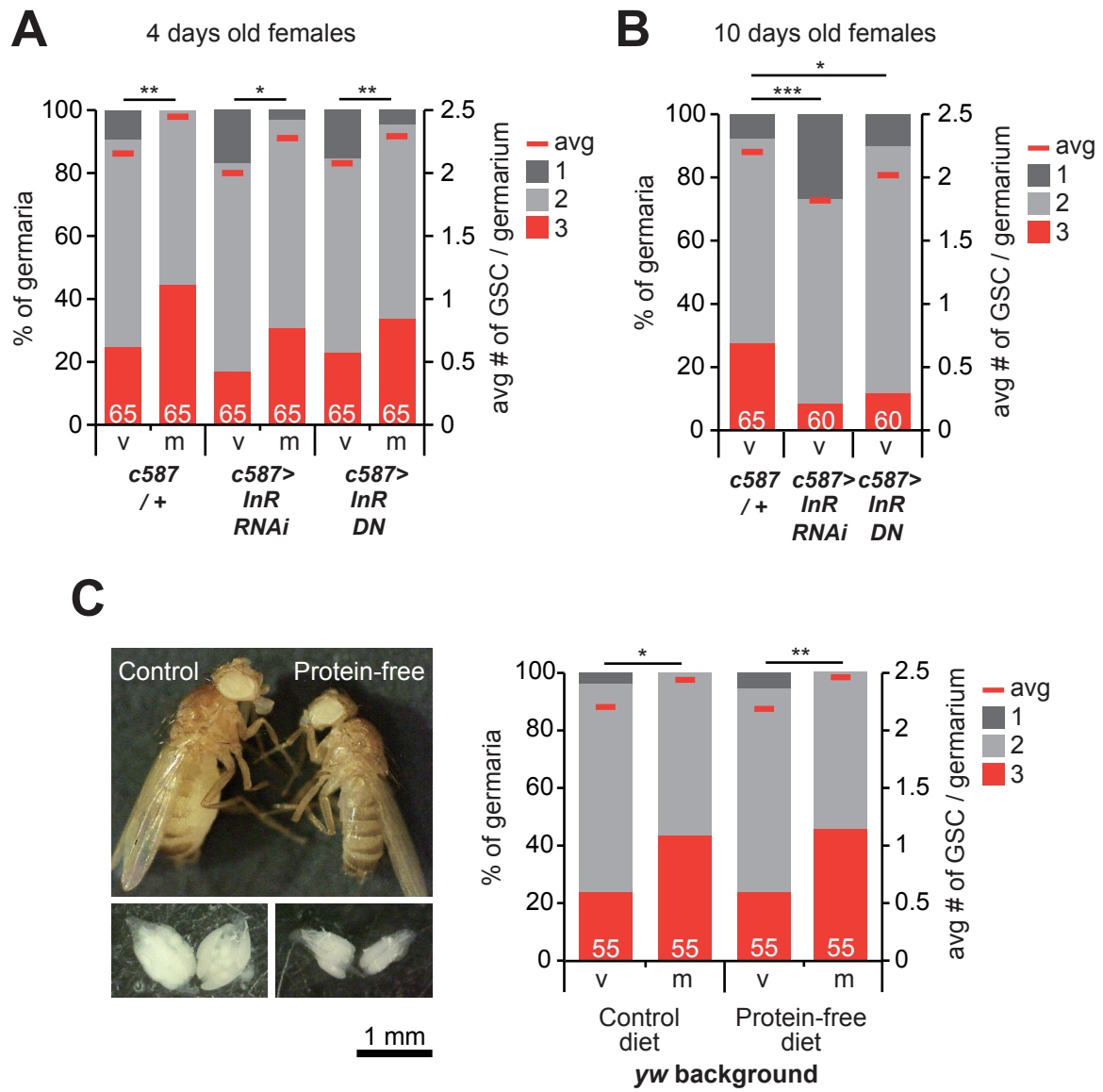

Supplement: S6 Fig — (A–C) Frequencies of germaria containing one, two, and three GSCs (left y axis), and average number of GSCs per germarium (right y axis) in virgin (v) and mated (m) female flies overexpressing an InR RNAi transgene or a dominant-negative form of InR in the ovarian somatic cells. Transgenes were driven by c587-GAL4. The female flies used were aged for 4 days (A) and 10 days (B). A mating-induced increase in GSC numbers occurred even in loss of InR females that showed age-dependent GSC loss (B). (C) Female flies fed protein-free diet also showed a significant increase in GSC number after mating. Female flies were fed grape juice-agar media with yeast paste (control) or not (protein-free diet). The numbers of germaria analyzed are shown inside bars. For statistical analysis, a Mann-Whitney U test was used. ***P ≤ 0.001, **P ≤ 0.01, *P ≤ 0.05. (PDF) [file pgen.1006123.s008.pdf]

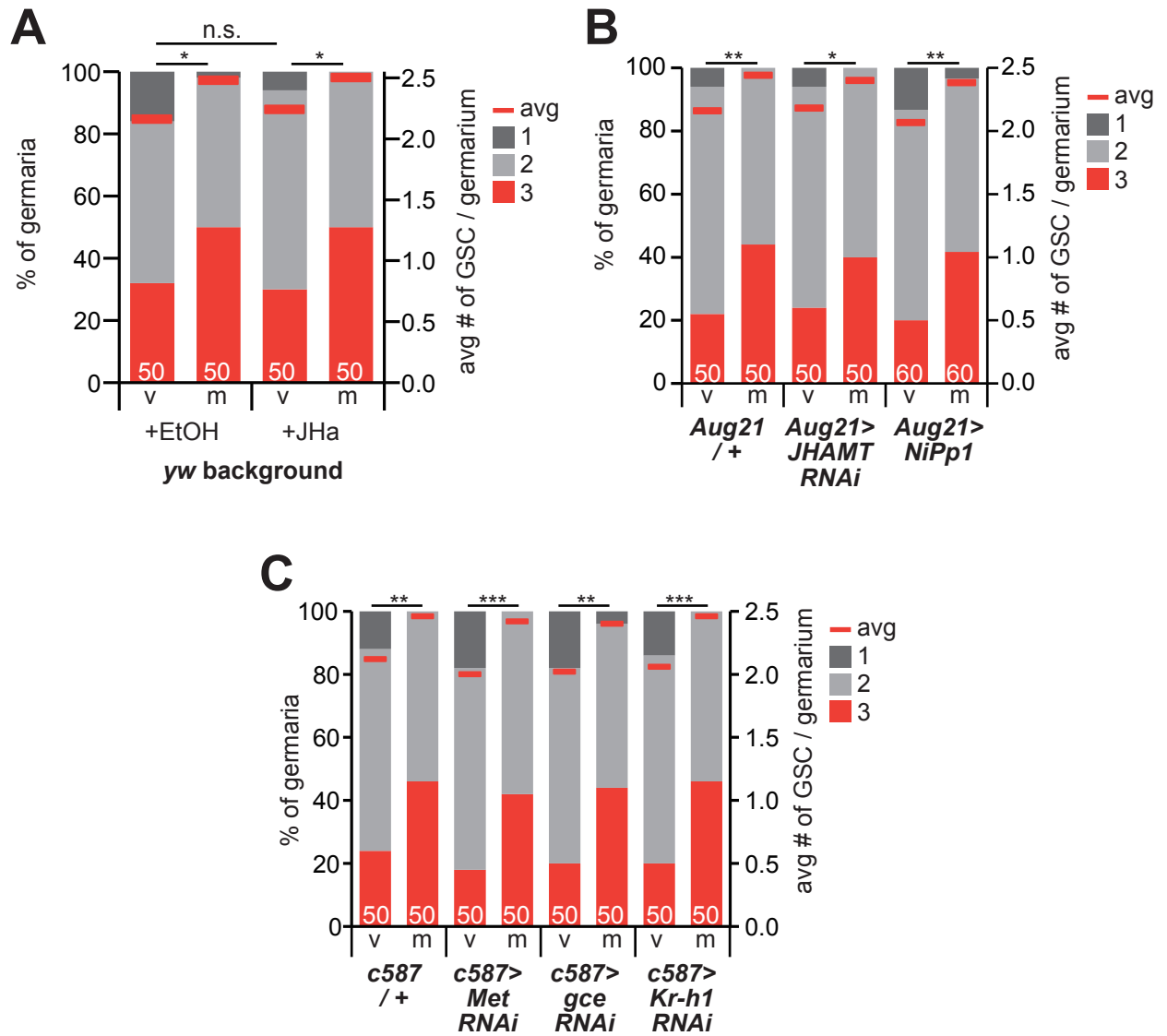

Supplement: S7 Fig — (A–C) Frequencies of germaria containing one, two, and three GSCs (left y axis), and average number of GSCs per germarium (right y axis) in virgin (v) and mated (m) female flies. (A) Oral administration of methoprene, a JH analogue (JHa) to virgin and mated females. (B) Blocking endogenous JH production by knocking down of juvenile hormone acid O-methyltransferase (JHAMT) or overexpression of the protein phosphatase inhibitor NiPp1 in the corpora allata (using Aug21-Gal4). (C) Knocking down JH receptor Met, gce or JH target Kr-h1 in the ovarian somatic cells (using c587-GAL4). The numbers of germaria analyzed are shown inside bars. For statistical analysis, a Mann-Whitney U test was used. ***P ≤ 0.001, **P ≤ 0.01, *P ≤ 0.05, n.s., non-significant (P > 0.05). (PDF) [file pgen.1006123.s009.pdf]
